# Realizing long-cycling silicon-based all-solid-state batteries with near-zero-stress variation

**DOI:** 10.1126/sciadv.aef3043

**Published:** 2026-08-26

**Authors:** Xu-Sheng Zhang, Jici Wen, Zhen-Zhen Shen, Xin Zhang, Jian-Xin Tian, Rui-Zhi Liu, Zehui Zhang, Kai-Xiang Zhou, Shuang-Yan Lang, Wen-Peng Wang, Sen Xin, Rui Wen, Yujie Wei, Li-Jun Wan, Yu-Guo Guo

**Affiliations:** ^1^ CAS Key Laboratory of Molecular Nanostructure and Nanotechnology, Beijing National Laboratory for Molecular Sciences (BNLMS), Institute of Chemistry, Chinese Academy of Sciences (CAS), Beijing 100190, People’s Republic of China.; ^2^ University of Chinese Academy of Sciences, Beijing 100190, People’s Republic of China.; ^3^ State Key Laboratory of Nonlinear Mechanics (LNM), Institute of Mechanics, CAS, Beijing 100190, People’s Republic of China.

## Abstract

Silicon anodes present a compelling alternative to lithium metal for all-solid-state batteries (ASSBs), offering high capacity without dendrite risks. However, their application is hindered by incomplete understanding of electro-chemo-mechanical (ECM) failure mechanisms in all-solid-state configurations. Through multiple in situ characterizations combining optical microscopy, atomic force microscopy, and pressure monitoring, this work uncovers fundamental stress-mediated degradation pathways in silicon-based ASSBs. Stress evolution—particularly in-plane strain mismatch and out-of-plane mechanical constraints—governs the dominant failure criterion, superseding traditional volume change metrics. This stress-dominated mechanism arises from the interplay between volume and modulus in constrained all-solid-state systems. Guided by these insights, complementary mitigation strategies were developed, including electrode/electrolyte modulus engineering to reduce interfacial stresses and elastic constraint design to accommodate mechanical fluctuations. The synergistic implementation achieves near-zero stress variation and breakthrough cycling stability (90.1% capacity retention after 5000 cycles). This work establishes a paradigm for high-energy-density batteries, shifting the design focus from volume accommodation to comprehensive stress management in all-solid-state systems.

## INTRODUCTION

All-solid-state batteries (ASSBs) have emerged as a transformative energy storage technology due to their inherent safety advantages from nonflammable solid-state electrolytes (SSEs) (*1*–*4*), yet their anode options remain constrained by fundamental material limitations. Silicon (Si) emerges as a game-changing anode material for ASSB applications, offering an exceptional theoretical capacity of 3590 mAh/g comparable to lithium (Li) metal while avoiding its dendrite-related safety concerns (*5*). Recent studies reveal that Si forms more stable interfaces with sulfide-based SSEs compared to Li metal anode, minimizing detrimental side reactions. The natural abundance and cost-effectiveness of silicon further enhance its practical appeal for large-scale ASSB deployment (*6*). Notably, silicon’s volume expansion—traditionally viewed as a drawback in liquid electrolyte systems—can be effectively accommodated in all-solid-state configurations through proper mechanical design (*7*). These combined advantages position Si as an ideal anode material for developing safe, high-energy-density ASSBs without the interfacial instability and safety limitations of Li metal systems.

The interfacial evolution and degradation mechanisms of Si anodes in ASSBs remain poorly understood compared to conventional liquid electrolyte systems. While substantial research efforts have been devoted to investigating Si anodes in liquid Li-ion batteries (*8*, *9*), limited studies have focused on their behavior in ASSBs. In liquid systems, the large volume changes (∼300%) during lithiation/delithiation lead to continuous solid-electrolyte interphase (SEI) reconstruction and consequent capacity fade (*10*, *11*). Although SSEs are expected to mitigate these stress variations, a comprehensive understanding of the electro-chemo-mechanical (ECM) effects and stress-induced failure mechanisms specific to Si-based ASSBs is still lacking.

The ECM behavior of Si anodes differs fundamentally between liquid and all-solid-state systems (*12*–*14*). In Si-based liquid batteries, research has primarily focused on mitigating volume changes and their associated failure mechanisms. In contrast, ASSBs present challenges due to solid-solid contact and mechanical constraints under high stack pressure. These conditions raise critical questions about whether traditional volume change metrics remain relevant for assessing performance in all-solid-state configurations. Moreover, while extensive in situ visualization research has been conducted in all-solid-state Li metal batteries (*15*–*17*), investigations for Si anodes remain scarce. Advanced in situ characterization techniques are urgently needed to elucidate the morphological evolution and ECM effects of Si anodes in ASSBs. An in-depth understanding of these processes is essential for optimizing the performance and reliability of Si-based ASSBs, particularly in addressing interface stability and stress management under operational conditions.

In this work, we systematically investigated the ECM evolution and failure mechanisms of Si-based anodes in ASSBs through an innovative combination of multiple in situ characterization techniques. By integrating in situ optical microscopy (OM), atomic force microscopy (AFM), and pressure monitoring (PM), we revealed the dynamic interplay between volume evolution, stress variation, and crack propagation in composite Si (CnSi) anodes during cycling. Our observations demonstrate that microscopic volume changes in CnSi particles during lithiation/delithiation (characterized by in situ AFM) induce pronounced pressure oscillations within individual charge-discharge cycles, along with progressive stress relaxation over prolonged cycling (monitored by in situ PM). Concurrently, extensive crack initiation and propagation are observed within SSEs (visualized by in situ OM), which ultimately contribute to ECM failure of ASSBs. We established stress variation—rather than volume change alone—as the critical failure criterion for Si-based ASSBs, reflecting the combined effects of volume evolution and modulus variation in these constrained solid-state systems. Through coupled computational and experimental analysis, we identified in-plane tensile stress as the fundamental driver of ECM failure. This stress is mainly induced by interfacial strain mismatch between the Si anode and SSEs, compounded by out-of-plane mechanical confinement. Guided by these insights, we developed targeted mechanical strategies to enhance structural integrity and cycling stability. These include (i) electrode/electrolyte modulus engineering to minimize interfacial strain mismatch and (ii) replacing rigid external constraints with elastic boundary conditions to accommodate stress variations and mitigate damage from out-of-plane expansion. This approach enables the realization of ASSBs with near-zero stress variation and exceptional cycling stability, achieving 99.3% capacity retention after 500 cycles, 98.6 after 2000 cycles, and 90.1% after 5000 cycles. These findings provide both fundamental insights into ECM failure mechanisms and practical design principles for developing durable high-energy-density ASSBs.

## RESULTS

### In situ ECM failure processes of ASSBs

**Fig. 1.**
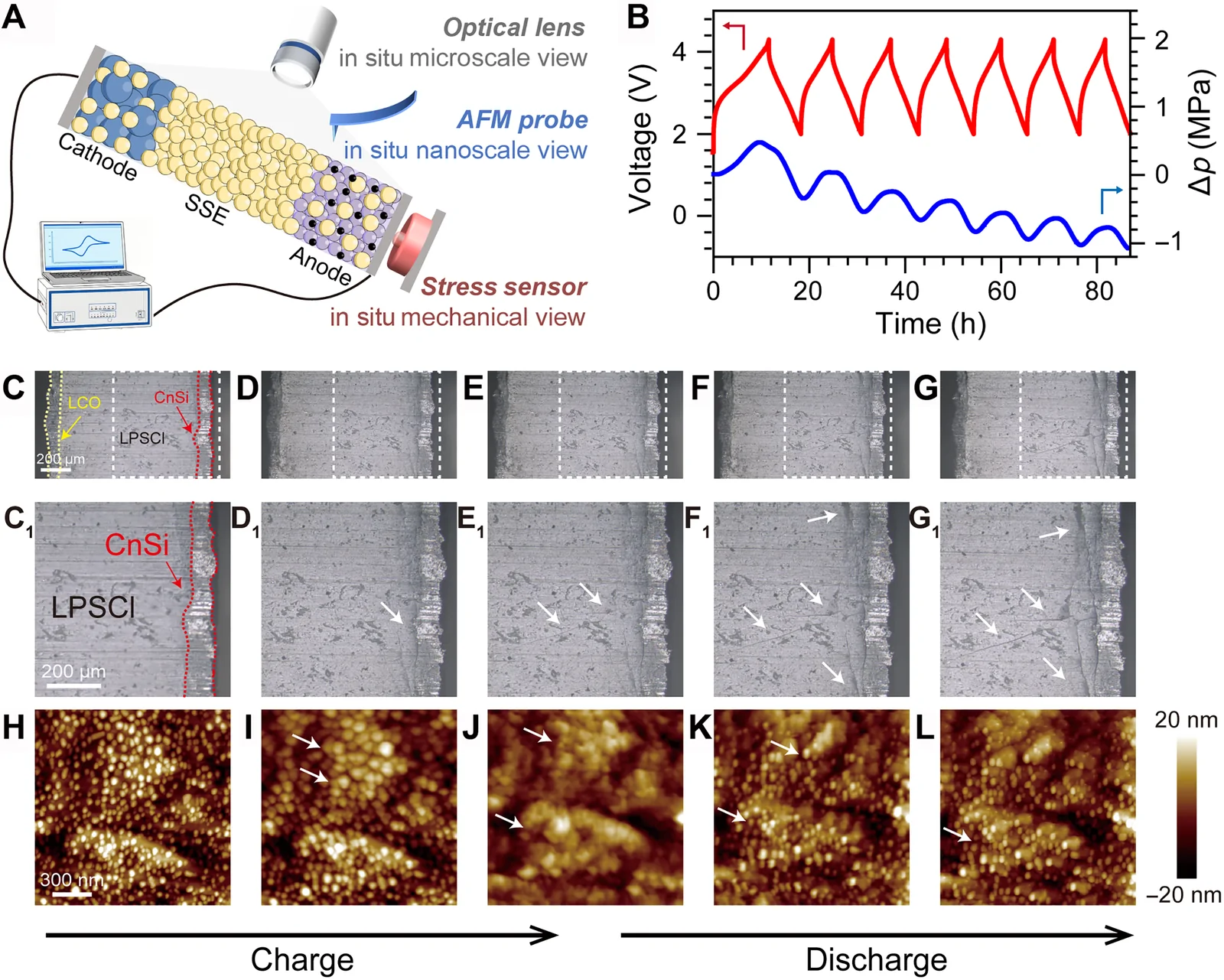
ECM failure evolution of Si-based anodes revealed by multiple in situ characterizations. (**A**) Scheme of the electrochemical cell for the multiple in situ characterizations, which includes in situ PM, in situ OM, and in situ AFM. (**B**) In situ PM curves and related charging/discharging curves of LiCoO_2_ (LCO)|Li_6_PS_5_Cl (LPSCl)|composite Si anodes (CnSi) ASSB at 0.1C. In situ OM images obtained at OCP (**C**), during charging (**D** and **E**) and during discharging (**F** and **G**) of LCO|LPSCl|CnSi ASSB at 0.1C. Magnified in situ OM images obtained at OCP (C_1_), during charging (D_1_ and E_1_) and during discharging (F_1_ and G_1_) of LCO|LPSCl|CnSi ASSB at 0.1C. The scale bars are 200 μm for (C) to (G) and (C_1_) to (G_1_). In situ AFM images obtained at OCP (H), during charging (**I** and **J**), and during discharging (**K** and **L**) of LCO|LPSCl|CnSi ASSB at 0.1C. The scale bars are 300 nm for (H) to (L), and the sweep direction is from bottom to top. h, hours.

**Fig. 2.**
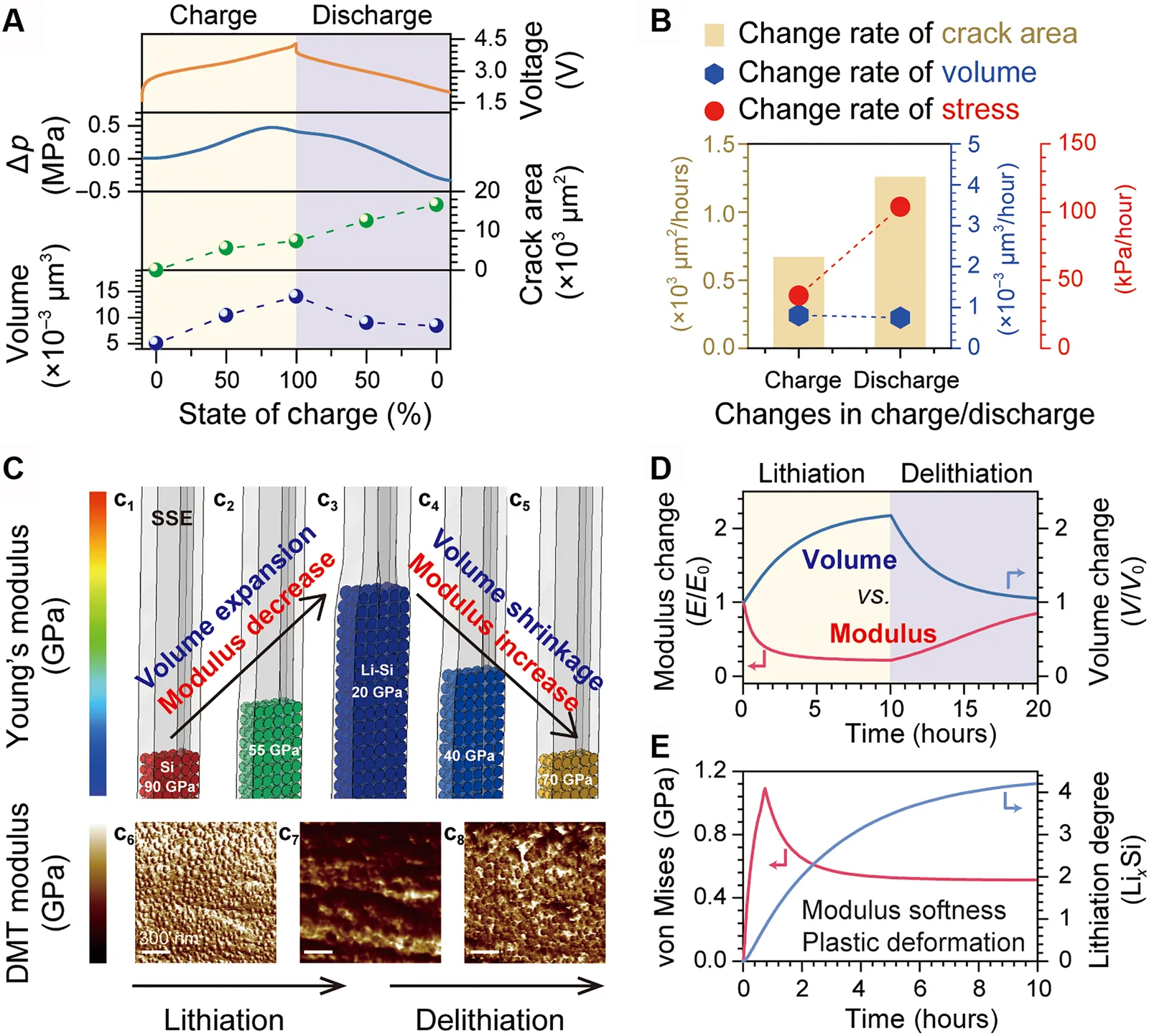
Coupled volume-modulus effects on stress evolution in Si-based ASSBs. (**A**) Stress changes from in situ PM, crack area changes from in situ OM, and volume changes from in situ AFM during the charging/discharging processes of LCO|LPSCl|CnSi ASSB at 0.1C. (**B**) Quantitative analysis of dynamic rates: projected crack area growth (1.26 × 10^3^ μm^2^/hour discharge versus 0.67 × 10^3^ μm^2^/hour charge), volume change (0.81 versus 0.75 × 10^−3^ μm^3^/hour), and stress variation (103.9 versus 38.7 kPa/hour), revealing modulus-dominated kinetics. (**C**) Integrated simulation-experimental validation: Top (C_1_ to C_5_): Simulation of Si-based anode deformation and modulus evolution at the anode-solid electrolyte interface (fixed perspective view). Bottom: In situ AFM DMT modulus images obtained at OCP (C_6_), at the end of first charge (C_7_), and at the end of first discharge (C_8_). The scale bars are 300 nm for (C_6_) to (C_8_), and the sweep direction is from bottom to top. (**D**) Evolution curves of volume and modulus of silicon particle during lithiation and delithiation with time. (**E**) Evolution curves of silicon particle lithiation ratio and von Mises stress with time.

To overcome Si’s limited conductivity, we fabricated composite Si anodes (CnSi) by blending nano-Si with Super P carbon and Li_6_PS_5_Cl (LPSCl) SSE (figs. S1 and S2). Within the CnSi anode, the active material and SSE were homogeneously mixed (figs. S3 to S5 and note S1). An ASSB was assembled with LiCoO_2_ (LCO) as cathode, Li_6_PS_5_Cl (LPSCl) as SSE, and CnSi as anode for in situ measurements. To elucidate the ECM coupling phenomena, we used complementary in situ characterization techniques to simultaneously monitor volume changes, stress evolution, and crack propagation during ASSB operation. As shown in Fig. 1A, in situ PM revealed stress fluctuations by recording pressure sensor data, while in situ OM and in situ AFM provided micro and nanoscale observations of CnSi during the ASSB charging/discharging. The coaxial pressure sensor enabled real-time monitoring of coupled stress evolution during electrochemical cycling, capturing the overall mechanical response of the ASSB. Before electrochemical testing, the assembled ASSB was allowed to stabilize under open circuit conditions to minimize baseline drift (fig. S6). The in situ PM data (Fig. 1B) revealed stress oscillations characterized by an increase during charging and a decrease during discharging, consistent with the deformation state of the CnSi electrode. This correlation indicates that CnSi predominantly governs the overall stress evolution in the ASSB. With prolonged cycling, a progressive stress relaxation was observed, which is primarily attributed to cracking-induced “pressure relaxation” within the system, where internal structural degradation manifests macroscopically as a gradual pressure drop. These measurements quantitatively capture the dynamic interplay between electrochemical reactions and mechanical stress evolution, as analyzed in detail in Fig. 2B. The surface of the LCO|LPSCl|CnSi ASSB at open circuit potential (OCP) was shown in Fig. 1C, with CnSi marked with red dash line and LCO marked with yellow dash line. No crack formation or morphological changes were detected before electrochemical cycling (fig. S7). The white dash line circled LPSCl|CnSi region in Fig. 1C was further magnified in Fig. 1C_1_. During the charging process, microcracks were detected near the LPSCl|CnSi interface (Fig. 1, D and D_1_). Following the completion of charge, small cracks emerged on the surface of LPSCl, as illustrated in Fig. 1, E and E_1_. Subsequently, during discharge, the previously formed small cracks widened and became more pronounced (Fig. 1, F and F_1_), ultimately leading to the observation of large cross-linked cracks at the end of discharge (Fig. 1, G and G_1_). The hierarchical crack morphology—consisting of both primary macroscopic cracks and secondary microcracks—was further evidenced by ex situ SEM (fig. S8 and note S2). Only rare and minor cracks were observed on the cathode side throughout the entire cycling process (fig. S9), indicating that mechanical failure at the cathode/electrolyte interface was substantially less severe than that at the anode side (note S3). Meanwhile, the in situ AFM analysis focused on the CnSi anode and illustrated the nanoscale evolution during charging and discharging. The AFM image in Fig. 1H displayed uniformly distributed nano CnSi particles in the anode at OCP. Upon charging, the lithiation of CnSi caused the nanoparticles to swell into larger formations (Fig. 1I), which subsequently merged into a mud-like amorphous morphology at the completion of the charge (Fig. 1J and fig. S10). During discharging, small particles emerged from the amorphous material, as depicted in Fig. 1 (K and L). These in situ methodologies provide a general framework for probing ECM coupling effect in constrained all-solid-state electrochemical systems (fig. S11)

Our integrated in situ analysis reveals the ECM evolution of Si-based anodes during ASSB operation. At different states of charges (SOCs), various parameters were monitored and quantitatively summarized in Fig. 2A and table S1: volume changes detected by in situ AFM, crack area evolution monitored by in situ OM, and stress variation recorded by in situ PM. Starting with the charging of LCO|LPSCl|CnSi ASSB, Li ions migrated from the LCO cathode to the CnSi anode, leading to the lithiation of CnSi. As CnSi underwent lithiation, nano-sized particles (5.11 × 10^−3^ μm^3^) exhibited volume expansion, increasing to 10.45 × 10^−3^ μm^3^ at 50% SOC and eventually merging into an amorphous Li-Si alloy with a volume of 14.03 × 10^−3^ μm^3^ at 100% SOC. The calculated volume expansion was ∼275%, close to its theoretical limit of 300% (*18*). The pronounced volume expansion induces internal stresses in the ASSB, leading to the nucleation and propagation of microcracks. The observed crack area grows from 5.58 × 10^3^ μm^2^ at 50% SOC to 7.37 × 10^3^ μm^2^ at 100% SOC. Subsequent discharging processes led to delithiation of the Li-Si alloy, resulting in the appearance of nano-sized Si particles on the muddy structure and a volume shrinkage (9.03 × 10^−3^ μm^3^ at 50% SOC and 8.48 × 10^−3^ μm^3^ at the end of charge). The volume shrinkage of the Si anode accompanied a reduction in stress, which caused the cracks to expand and become more pronounced (16.63 × 10^3^ μm^2^ at the end of charge). The continual growth of the cracks further compromised the ion conducting pathways, ultimately leading to the ECM failure of the Si-based ASSBs.

### Stress-dominated ECM failure mechanism in ASSBs

The quantitative analysis of the ECM failure in Si-based ASSBs reveals a fundamental shift in degradation metrics compared to liquid systems, revealing mechanical stress response rather than volume change as the primary driver of ECM degradation for ASSBs. While volume change rates remain similar during charge (0.81 × 10^−3^ μm^3^/hour) and discharge (0.75 × 10^−3^ μm^3^/hour) at 0.1C, the projected crack area evolution, measured via in situ OM within a field of view of 800 μm by 800 μm, exhibits markedly different kinetics—accelerating to 1.26 × 10^3^ μm^2^/hour during discharge versus 0.67 × 10^3^ μm^2^/hour during charge (Fig. 2B). This discrepancy demonstrates that traditional volume-based failure metrics in liquid systems are inadequate for all-solid-state systems. Crucially, pressure evolution shows superior correlation with damage progression: the stress decreases at an average rate of 103.9 kPa/hour during discharge compared to an average increase rate of 38.7 kPa/hour during charge (fig. S12), highlighting the substantially higher rate during discharge. The capacity exhibited a gradual decline during cycling (fig. S13), with the degradation rate closely correlated to the corresponding stress variations (fig. S14 and table S2). These findings establish that in the constrained environment of ASSBs, mechanical stress response rather than volume change serves as the primary driver of ECM degradation, necessitating a paradigm shift from liquid-system evaluation approaches to stress-dominated failure analysis for accurate assessment of Si-based anode durability in ASSBs.

The observed decoupling between volumetric and mechanical degradation pathways highlights the ECM coupling in solid-state architectures. The twofold greater crack area growth during discharge, despite comparable volume change rates, underscores how stress dominates the failure process. This stress-centric failure mechanism fundamentally differs from liquid systems, where volume changes primarily govern degradation. These insights provide critical guidance for developing durable solid-state battery designs, emphasizing the need to monitor and mitigate stress evolution rather than simply accommodate volume changes as in conventional battery systems.

The recognition of stress as a primary determinant of ECM failure in all-solid-state systems necessitates a fundamental re-examination of its governing parameters. Through coupled mechanical simulations and in situ AFM modulus mapping, we systematically investigated the synergistic deformation-stress evolution in composite Si anodes during electrochemical cycling. Volumetric evolution and mechanical modulus were found to be pivotal factors governing the ECM response: (i) pronounced volumetric changes, especially full lithiation in Si materials, induce severe deformation and stress in SSEs, and (ii) concurrent modulus softening and plastic flow of Si during lithiation effectively mitigate severe stress states and damage in SSEs. These findings challenge the conventional volume-centric paradigm and instead highlight the critical, yet overlooked, role of mechanical modulus in governing ECM stress evolution.

As illustrated in Fig. 2C, the original modulus of Si at OCP was 90 GPa (Fig. 2C_1_) (*19*), which experienced volume expansion during lithiation along with modulus decreased to 20 GPa as Li-Si alloy (Fig. 2, C_2_ and C_3_). During delithiation, volume shrinkage and modulus increase can be detected (Fig. 2, C_4_ and C_5_), implying the interplay of volume variation and modulus changes. In addition to offering insights into the morphological evolution, in situ AFM also provided Derjaguin-Muller-Toporov (DMT) modulus information, which can further prove the interplay between volume and modulus experimentally. The DMT modulus of pristine CnSi at OCP was ∼3.3 GPa, as depicted in Fig. 2C_3_ (morphology depicted in Fig. 1H). Upon charging, CnSi underwent lithiation, transforming into Li-Si alloy and causing a decrease in DMT modulus to 1.2 GPa, as shown in Fig. 2C_7_ (morphology displayed in Fig. 1J). Subsequently, during discharging, the Li-Si alloy delithiated, with a slight increase in DMT modulus to 2.3 GPa (Fig. 2C_8_, morphology in Fig. 1L). The stress evolution in Si-based anodes is governed by the dynamic interplay between competing volumetric and mechanical transformations during electrochemical cycling. As illustrated in Fig. 2D, this process involves two counteracting mechanisms: substantial volumetric expansion that generates compressive stresses and concurrent modulus reduction that enables stress relaxation through mechanical softening. Finite element simulations (Fig. 2E) identify three distinct stress regimes: (i) initial elastic stress buildup in high-modulus pristine Si (90 GPa), (ii) stress stabilization through the formation of softened amorphous Li-Si phases, and (iii) plastic-flow dominated deformation at higher lithiation degrees. During delithiation, the reverse process occurs, with volume contraction and modulus recovery creating complex stress patterns that contribute to cumulative damage.

Our comprehensive in situ characterization reveals that ECM failure in Si-based ASSBs is governed by the coupled effects of volume changes and modulus evolution. Unlike conventional liquid systems where volume expansion primarily dictates mechanical degradation, solid-state Si-based anodes exhibit complex stress-strain responses dominated by phase-dependent modulus changes (discussion S1) and plastic deformation. Crucially, we demonstrate that stress evolution—modulated by dynamic modulus variations and plastic deformation—provides a more accurate and comprehensive metric for assessing ECM failure than volume change alone. Despite the potential influence of interfacial chemical degradation (*20*), the stress-dominated failure mechanism established in this work remains valid and can be extended from composite electrodes to Si-only configurations (figs. S15 to S17, table S3, and note S4). These findings necessitate a fundamental reevaluation of design strategies for Si-based anodes, shifting focus from volume accommodation to active modulus engineering and stress management in ASSB architectures.

### ECM failure mechanism: In-plane tensile stress

**Fig. 3.**
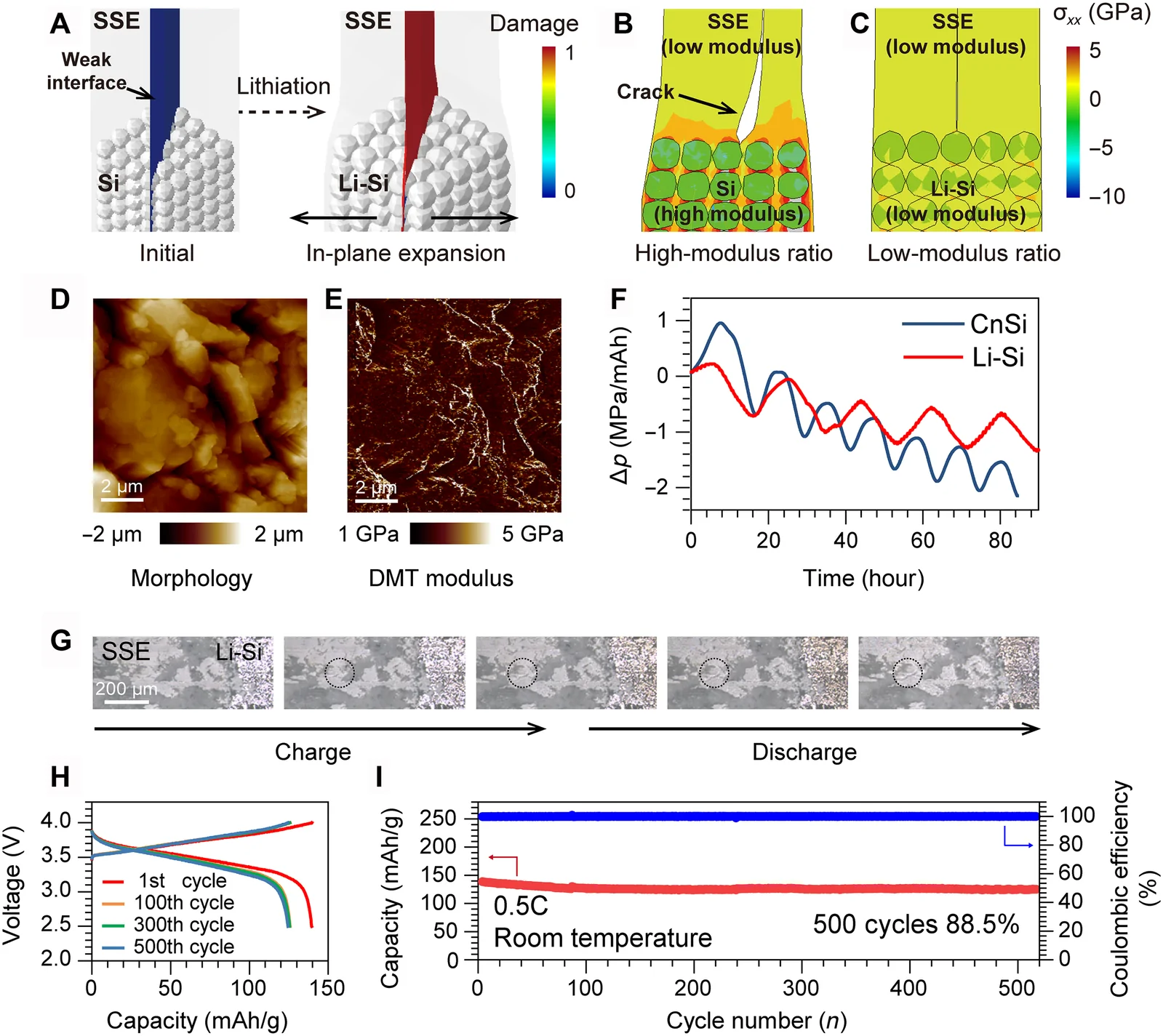
In-plane expansion induced mode I fracture mechanisms in Si-based ASSBs. (**A**) Finite element model illustrating the local microstructure at the electrode particle/SSE interface and damage evolution simulation demonstrating crack initiation and propagation caused by in-plane expansion of Si anodes during lithiation. (**B**) High-modulus Si anode paired with low-modulus SSE generates substantial tensile stresses (up to 5 GPa) at interfaces, leading to prominent crack formation (black arrow). (**C**) Low-modulus Li-Si alloy anode with low-modulus SSE configuration mitigates stress concentration and retard crack propagation. (**D**) AFM images showing the morphology of Li-Si alloy anodes. (**E**) AFM DMT modulus image of Li-Si alloy anodes. (**F**) In situ PM curves of ASSBs with CnSi as anode and Li-Si alloy as anode. (**G**) In situ OM images obtained at OCP, during charging, and discharging of LCO|LPSCl|Li-Si ASSB. (**H**) Charge and discharge curves of LCO|LPSCl|Li-Si ASSBs at 0.5C. (**I**) Cycling performances of LCO|LPSCl|Li-Si ASSBs at 0.5C.

Through coupled computational and experimental investigations, we elucidate the fundamental ECM failure mechanisms in Si anodes and SSEs within ASSBs. Lithiation-induced expansion in Si anodes generates complex stress states within the SSEs: (i) in-plane tensile stresses arising from constrained lateral expansion and (ii) out-of-plane compressive stresses under stack pressure confinement. To elucidate the ECM failure mechanism, we conducted dynamic mechanical simulations on a representative volume element (RVE) of the composite anode-SSE interface (Fig. 3A, left). A weak interface (marked in Fig. 3A left), with cohesive strength substantially lower than the SSE yield strength, was introduced to model the fracture-susceptible region. Using a coupled chemo-mechanical framework, we concurrently simulated lithium-ion diffusion, structural deformation, and damage evolution during cycling. The simulations reveal that the large in-plane expansion of silicon during lithiation induces substantial mismatch between the Si anode and the SSE, generating high tensile stresses (σ_xx_ ≈ 5 GPa) (Fig. 3A, right). These high tensile stresses challenge interfacial imperfections such as weakly bonded regions, preexisting flaws, or grain boundaries drive crack initiation, growth, coalescence, and propagation (see for example the mode I type of fracture marked in Fig. 3B). Experimentally, cracking within the SSE correlates with degradation in both mechanical integrity (e.g., loss of stack pressure) and electrochemical performance (e.g., capacity fading), highlighting the critical role of stress management in the design of mechanically robust ASSBs.

To address ECM failure arising from mismatch strain between the Si anode and SSEs in ASSBs, we propose a strain-mismatch mitigation strategy based on control of the modulus ratio of anode to SSE via pre-lithiation. A reduced anode/SSE modulus ratio effectively suppresses tensile stress accumulation within the SSE layer (Fig. 3, B and C), thereby enhancing interfacial mechanical stability. The formation of mechanically softened Li-Si alloys, as characterized by AFM DMT imaging (Fig. 3, D and E, and discussion S2), results in a lower elastic modulus and yield strength. This mechanical softening allows the expansion of Si particles to be more effectively constrained by the surrounding SSE, while pre-lithiation simultaneously reduces the magnitude of volumetric expansion. Together, these effects alleviate interfacial mismatch strain and mitigate tensile stress development in the SSE. This integrated approach achieves substantial performance improvements, including a substantial reduction in interfacial stress fluctuations (8.73 kPa/hour during charging and 65.3 kPa/hour during discharging) relative to conventional silicon anodes (Fig. 3F, fig. S18, and table S4), significant decrease of observable cracking during cycling (Fig. 3G), and long-term electrochemical stability with 88.5% capacity retention after 500 cycles at 0.5C (Fig. 3, H and I). These findings establish modulus engineering as a fundamental design paradigm for developing durable, high-capacity anodes in solid-state battery systems.

The demonstrated approach offers a generalizable framework for overcoming interfacial degradation challenges in next-generation energy storage technologies. While this study primarily focuses on adjusting the anode modulus through controlled pre-lithiation of silicon, we note that equivalent modulus ratio optimization could alternatively be achieved by engineering the SSE’s mechanical properties—an important direction for future research. Future studies can explore complementary strategies such as tailoring solid electrolyte composition and microstructure to achieve optimal modulus ratios, potentially enabling further improvements in mechanical stability and electrochemical performance. This dual approach—engineering both anode and electrolyte properties—may provide additional degrees of freedom for designing robust, high-energy-density solid-state battery systems, particularly for alloying-type anode materials undergoing substantial volume changes during operation.

### ECM failure mechanism: Out-of-plane restriction forces

**Fig. 4.**
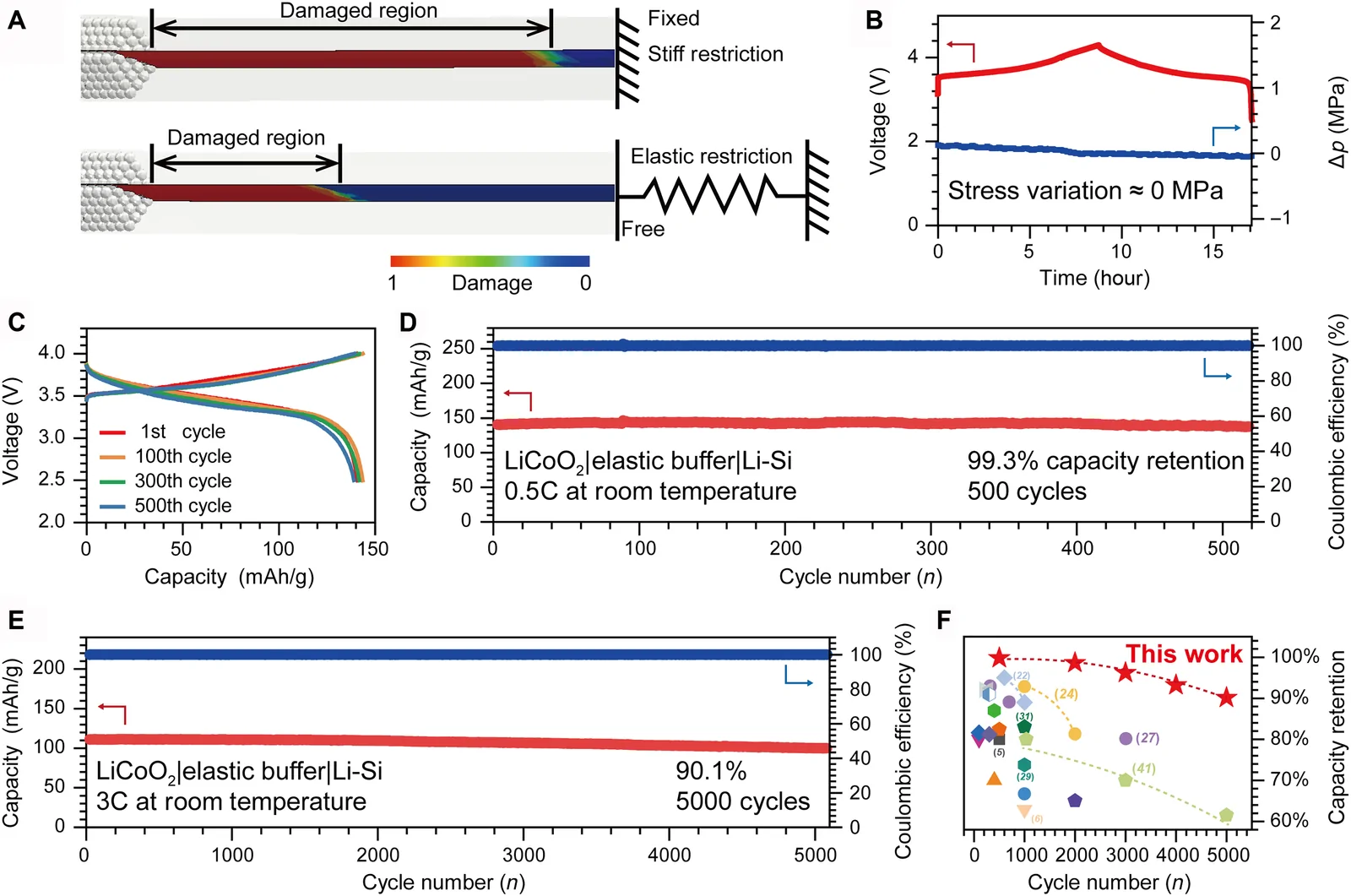
Out-of-plane restriction effects on mode I fracture mechanisms in Si-based ASSBs. (**A**) Comparative damage evolution simulations reveal severe damage due to unaccommodated out-of-plane expansion under stiff restriction (top) and reduction in damage degree through stress dissipation under elastic restriction (bottom). (**B**) In situ PM curves and related charging/discharging curves of LCO|Elastic buffer|Li-Si ASSB at 0.1C. (**C**) Charge and discharge curves of LCO|Elastic buffer|Li-Si ASSBs at 0.5C. (**D**) Cycling performances of LCO|Elastic buffer|Li-Si ASSBs at 0.5C. (**E**) Cycling performances of LCO|Elastic buffer|Li-Si ASSBs at 3C. (**F**) Performance comparison with state-of-the-art ASSBs (colored symbols) from peer-reviewed literature (*5*, *6*, *21*–*38*). Our elastic buffering strategy (red star) achieves record capacity retention under long-cycling condition.

While modulus engineering of the anode and SSEs can mitigate in-plane tensile stresses induced by electrode expansion, the out-of-plane stress generated during anode expansion also plays a critical role in ECM failure of Si-based ASSBs. In conventional ASSB configurations, the cell is subjected to rigid mechanical constraints in the normal direction, severely restricting out-of-plane expansion. Confined normal expansion exacerbated the electrode film’s in-plane expansion, leading to mode I fracture and significant SSE damage (Fig. 4A_1_). Consequently, addressing out-of-plane stress is essential for ECM stability.

Simulation results reveal that relaxing the constraints by placing low-level pressure can substantially mitigate out-of-plane expansion-induced damage (Fig. 4A_2_). Guided by this finding, we implemented an ASSB system incorporating an elastic buffering—a concept that can be further simplified in future studies through the development of elastic buffer interlayers or elastic SSEs. By using such an elastic cushion, we achieved an ASSB system with near-zero stress variation (less than 5 kPa/hour during both charge and discharge; fig. S19 and table S4), which was effectively maintained over subsequent cycles (fig. S20), leading to significantly improved ECM stability (Fig. 4B).

Under these near-zero stress conditions, the ASSB exhibits exceptional electrochemical performance, delivering a high reversible capacity of 140.4 mAh/g with remarkable cycling stability—retaining 143.7, 142, and 139.4 mAh/g after 100, 300, and 500 cycles, respectively (Fig. 4C). The elastic-buffered ASSB demonstrates outstanding long-term cyclability, maintaining 99.3% capacity retention after 500 cycles (Fig. 4D). Even under high-rate (3C) conditions, the cell exhibits superior performance (fig. S21), with 98.6% retention after 2000 cycles and 90.1% retention after 5000 cycles (Fig. 4E). The proposed elastic buffer strategy not only surpasses the performance of many reported ASSBs (*5*, *6*, *21*–*38*) in terms of capacity retention and cycle life but also demonstrates exceptional stability under high-rate conditions—a critical requirement for practical applications (Fig. 4F, fig. S22, and table S5). Compared to rigidly constrained systems that often suffer from rapid degradation due to stress accumulation, our approach effectively minimizes mechanical degradation, enabling stable long-term operation. These results highlight the immense potential of elastic mechanical design in advancing high-energy-density ASSBs, particularly for alloying-type electrodes such as Si-based anodes where volume expansion remains a major challenge.

Our results demonstrate that reducing the out-of-plane restriction force (exemplified here by the use of a spring as a model tool) effectively suppresses mechanical degradation and crack propagation. It should be emphasized that the spring serves merely as an experimental tool to validate the underlying mechanism, rather than constituting the mitigation strategy itself. Looking ahead, further optimization of elastic buffer materials—such as tunable polymer-ceramic composites or gradient-modulus SSEs—could enhance both mechanical compliance and ionic conductivity. In addition, integrating this strategy into large-format pouch or prismatic cells will be essential for commercialization. This work provides a foundational framework for addressing ECM failures in ASSBs, paving the way for more robust and durable solid-state batteries.

## DISCUSSION

In summary, this study provides fundamental insights into ECM failure mechanisms of Si-based anodes in ASSBs through a comprehensive experimental and computational approach. By employing a suite of in situ characterization techniques, we have established that mechanical stress evolution, rather than volumetric changes alone, serves as the determining factor for electrode stability in constrained solid-state systems. The identification of in-plane tensile stress and out-of-plane constraints as the primary failure driver has led to the development of two effective mitigation strategies: optimized electrode/electrolyte modulus matching and innovative elastic constraint design. These approaches collectively address the critical stress-related challenges, enabling the realization of high-performance ASSBs with exceptional cycling stability. The findings of this work not only advance our fundamental understanding of ECM interactions in solid-state battery systems but also establish practical design principles for developing durable, high-energy-density energy storage devices. The stress management strategies presented here offer a paradigm for overcoming the long-standing challenges associated with Si-based anodes in solid-state configurations, paving the way for next-generation battery technologies with improved performance and reliability. Future research directions may focus on further optimization of elastic constraint materials and exploration of these principles in large-format cell designs to facilitate practical implementation.

## MATERIALS AND METHODS

### Materials preparation

The composite cathodes were fabricated by thoroughly mixing LiCoO_2_ (Canrd Technology Co. Ltd) and Li_3_InCl_6_ (Hefei Kejing Corp.) at a 60:40 weight % (wt %) ratio via mortar-and-pestle grinding for 30 min in an argon-filled glovebox [MIKROUNA Super 1220/750; H_2_O < 0.1 parts per million (ppm), O_2_ < 0.1 ppm]. Composite nano-Si anodes were synthesized by ball-milling nano-Si (30 nm; 99.9%, Innochem), Li_6_PS_5_Cl (Hefei Kejing Corp.), and Super P (99.0+%, Alfa Aesar) at 60:30:10 wt % in an argon-atmosphere milling jar (400 rpm, 4 hours). Li-Si alloy anodes were prepared by prelithiating nano-Si powders (30 nm, 99.9%) with Li metal. All procedures were conducted under strict inert conditions to prevent material degradation.

### ASSB assembly

The ASSB pellets were fabricated through a sequential cold-pressing protocol under inert atmosphere. First, 80 mg of Li_6_PS_5_Cl solid electrolyte powder (Hefei Kejing Corp.) was uniaxially pressed at 300 MPa to form a dense separator layer. The composite cathode (LiCoO_2_:Li_3_InCl_6_ = 60:40 wt %) and anode (composite nano-Si or Li-Si alloy) were then symmetrically assembled on either side of the electrolyte layer. The multilayer structure was finally consolidated under 400 MPa pressure for 10 min in an argon-filled glovebox (MIKROUNA Super 1220/750; H_2_O < 0.1 ppm, O_2_ < 0.1 ppm), ensuring intimate interfacial contact while maintaining mechanical stability. This optimized protocol yielded robust ASSB pellets with uniform thickness and well-integrated electrode-electrolyte interfaces. The entire assembly process was performed without atmospheric exposure to prevent electrolyte decomposition.

### In situ PM characterization

The pressure evolution during cycling was monitored using a custom-built PM system featuring an integrated piezoresistive pressure sensor coaxially aligned with the cell stack (Fig. 1A). Before electrochemical testing, the assembled cell was allowed to stabilize under open circuit conditions for 36 hours to minimize baseline drift. During the final 6 hours of this resting period, the baseline pressure fluctuation was confined within ±0.025 MPa (fig. S6). The galvanostatic cycling of both LCO|LPSCl|CnSi and LCO|LPSCl|Li-Si ASSBs was performed using a NEWARE battery test system (CT-4008Tn-5V50mA-164). The sensor’s linear response range and temperature compensation (25° ± 0.5°C) enabled precise correlation between electrochemical processes and mechanical stress evolution.

### In situ OM characterization

The morphological evolution of the LCO|LPSCl|CnSi ASSB during cycling was investigated using an OLYMPUS OLS-4000 laser scanning confocal microscope equipped with a 20× objective lens [LUCPLFLN 20×, numerical aperture (NA) = 0.4]. Before testing, the ASSB pellet was mounted in a custom-designed electrochemical cell with optical access, and the observation surface was carefully polished to achieve flatness for optimal imaging quality. The microscope was integrated with a NEWARE battery test system (CT-4008Tn-5V50mA-164) to enable synchronous electrochemical cycling and morphological observation.

### In situ AFM characterization

The morphological and mechanical evolution of the LCO|LPSCl|CnSi ASSB during cycling was investigated using a Bruker Dimension Icon AFM system integrated with a NEWARE battery tester (CT-4008Tn-5V50mA-164) in an argon-filled glovebox (MIKROUNA Super 1220/750; H_2_O < 0.1 ppm, O_2_ < 0.1 ppm). Before testing, the ASSB pellet surface was carefully polished to sub-micron flatness to ensure imaging stability. AFM measurements were conducted in PeakForce Tapping mode at a scan rate of 1.0 Hz and with 256 × 256 pixel resolution. The system simultaneously captured topographical changes and Young’s modulus evolution using the DMT model. All acquired images were processed using NanoScope Analysis software with flattening and Fast Fourier Transform (FFT)-based noise reduction algorithms to maintain accurate surface representation while enhancing feature contrast.

### Electrochemical characterization

The electrochemical performance of the ASSB pellets was evaluated using a custom mold cell (Beijing Zhongke Wanyuan Technology Co. Ltd.) connected to a NEWARE battery test system (CT-4008Tn-5V50mA-164) under controlled conditions (25° ± 0.5°C). The composite cathode areal loading was controlled to be around 10 mg/cm^2^. The operating pressure for ASSBs was controlled to be around 100 MPa. For stress accommodation studies, ASSBs incorporating elastic buffer layers were assembled in a modified mold featuring integrated springs to mitigate mechanical stress fluctuations during cycling. The spring constant was 568.4 N/mm. This innovative design enabled real-time stress compensation while maintaining stable electrode-electrolyte interfaces. All assembly processes were performed under argon atmosphere to prevent electrolyte decomposition.

### Ex situ characterizations

Post-cycling structural and morphological analyses were systematically performed to investigate the electrochemical degradation mechanisms. X-ray diffraction measurements were conducted using a Rigaku SmartLab diffractometer with Cu Kα radiation, operating under vacuum to prevent air exposure of sensitive components. Patterns were collected from 10° to 80° (2θ) at a scanning rate of 5°/min. Raman spectroscopy was performed on a Princeton SP2356/PYL-100BX system equipped with a 532-nm laser and 20× objective lens (NA = 0.4). Morphological characterization was carried out using field-emission scanning electron microscopy (SEM) (JEOL JSM-6701F) and SEM specimen mounts [Zhongjingkeyi (Beijing) Film Technology Co. Ltd]. All ex situ samples were carefully transferred under argon atmosphere to preserve their native states.

### First-principles computational analysis

We conducted comprehensive density functional theory calculations to investigate the fundamental mechanical properties of the electrode and electrolyte materials. All calculations were performed using the Vienna Ab initio Simulation Package with projector augmented wave pseudopotentials and the Perdew–Burke–Ernzerhof generalized gradient approximation (PBE-GGA) exchange-correlation functional. The elastic properties were systematically evaluated through the stress-strain method, where we applied six independent strain components to calculate the complete elastic constant tensor. These calculations enabled us to derive key mechanical parameters including the bulk modulus (B), shear modulus (G), Young’s modulus [E = 9BG/(3B+G)], and Poisson’s ratio [ν = (3B-2G)/(6B+2G)] through the Voigt-Reuss-Hill approximation. For enhanced data analysis and visualization, we used the ELATE platform (*39*) to analyze the full elastic tensors and their anisotropic characteristics.

### Mechanical simulations

For model construction, ABAQUS CAE (DS Simulia Corp., 2020) was used to generate a composite structural model of the electrode-SSE interface. Both the electrolyte material and the SSE were meshed with three-dimensional tetrahedral elements. The interface was discretized and was represented by using cohesive elements, which are capable of capturing potential delamination and fracture behaviors. A representative volume element (RVE) was extracted from the electrode thin film by selecting a square region of 5 μm in length within the in-plane direction and slicing through the thickness direction at the location where fracture predominantly occurs, namely, the composite anode-SSE layer. The anode was idealized as a homogeneous distribution of spherical silicon particles with an average radius of 0.49 μm and encapsulated by the SSE. In the model, the anode thickness was set to 100 μm and the SSE layer thickness to 900 μm. A weak interface was introduced within the composite structure, with cohesive strength substantially lower than the yield strength of the SSE, representing the region most vunerable to fracture. The simulation focused on the volumetric change of the silicon anode during (de-)lithiation and the resulting damage evolution within the composite anode-SSE structure. A coupled chemo-mechanical framework was adopted, in which lithium-ion diffusion, structural deformation, and damage accumulation during charge-discharge cycling were simulated concurrently. The governing equations follow the mechanical-diffusion coupling theory, and the numerical implementation was carried out via a user-defined element subroutine within the ABAQUS environment to enable fully coupled RVE simulations, following the reported articles (*40*, *41*).

## Supplementary Materials

### This PDF file includes:

Figs. S1 to S35 Tables S1 to S18 Notes S1 to S4 Discussions S1 and S2 References
