# Supplementary material for: Realizing long-cycling silicon-based all-solid-state batteries with near-zero-stress variation
Source: Sci Adv. 2026 Aug 26;12(35):eaef3043. doi: 10.1126/sciadv.aef3043 (PMC13510668; doi:10.1126/sciadv.aef3043)
Supplement: Supplementary file 1 [file sciadv.aef3043_sm.pdf]

Science Advances
AAAS

# Supplementary Materials for

## Realizing long-cycling silicon-based all-solid-state batteries with near-zero-stress variation

Xu-Sheng Zhang *et al.*

Corresponding author: Rui Wen, ruiwen@iccas.ac.cn; Yujie Wei, yujiewei@lnm.imech.ac.cn; Yu-Guo Guo, ygguo@iccas.ac.cn

*Sci. Adv.* **12**, eaef3043 (2026)
DOI: 10.1126/sciadv.aef3043

**This PDF file includes:**

Figs. S1 to S35
Tables S1 to S18
Notes S1 to S4
Discussions S1 and S2
References

# Supplementary Figures

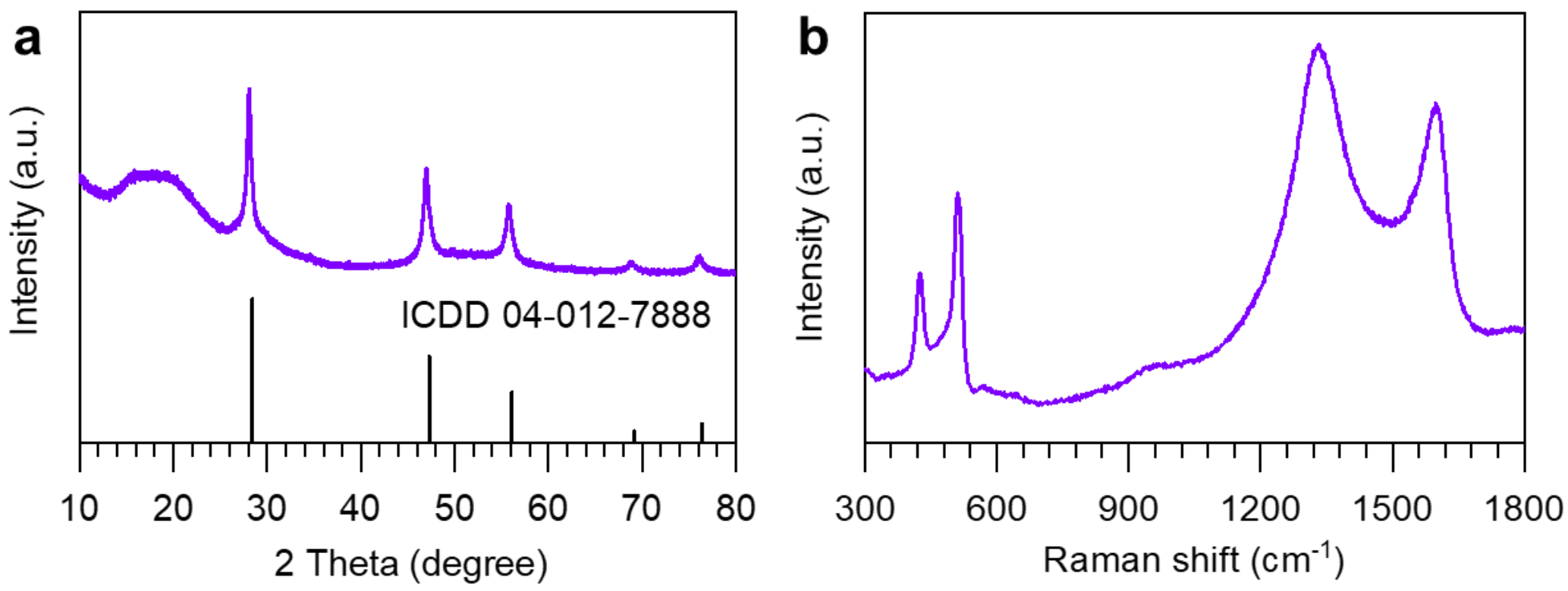


**Fig. S1. Characterization of the composite Si (CnSi) anode.** (a) X-ray diffraction (XRD) patterns and (b) Raman spectrum of the CnSi composite.

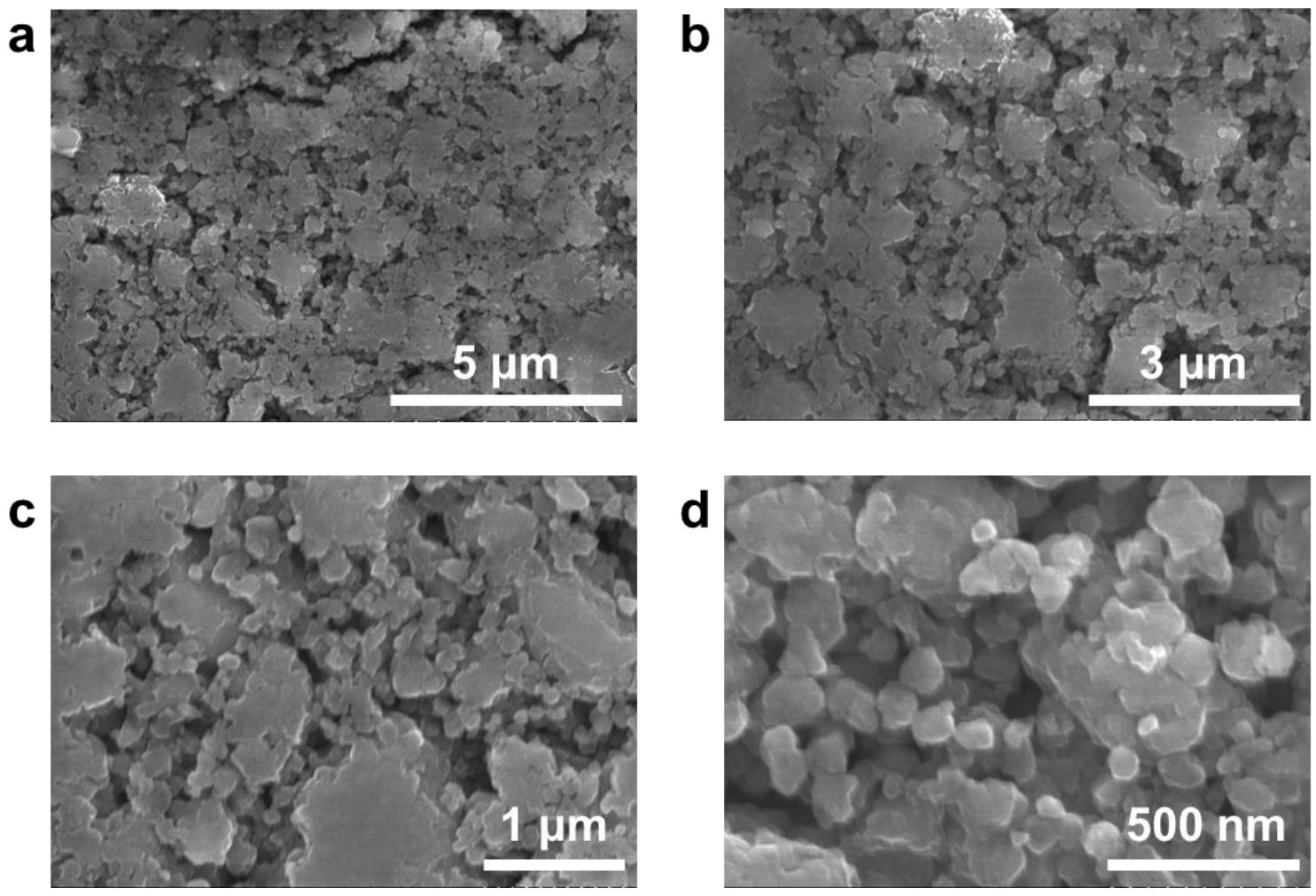


**Fig. S2. Morphology of the CnSi composite anode.** Representative scanning electron microscopy (SEM) images of the CnSi composite.

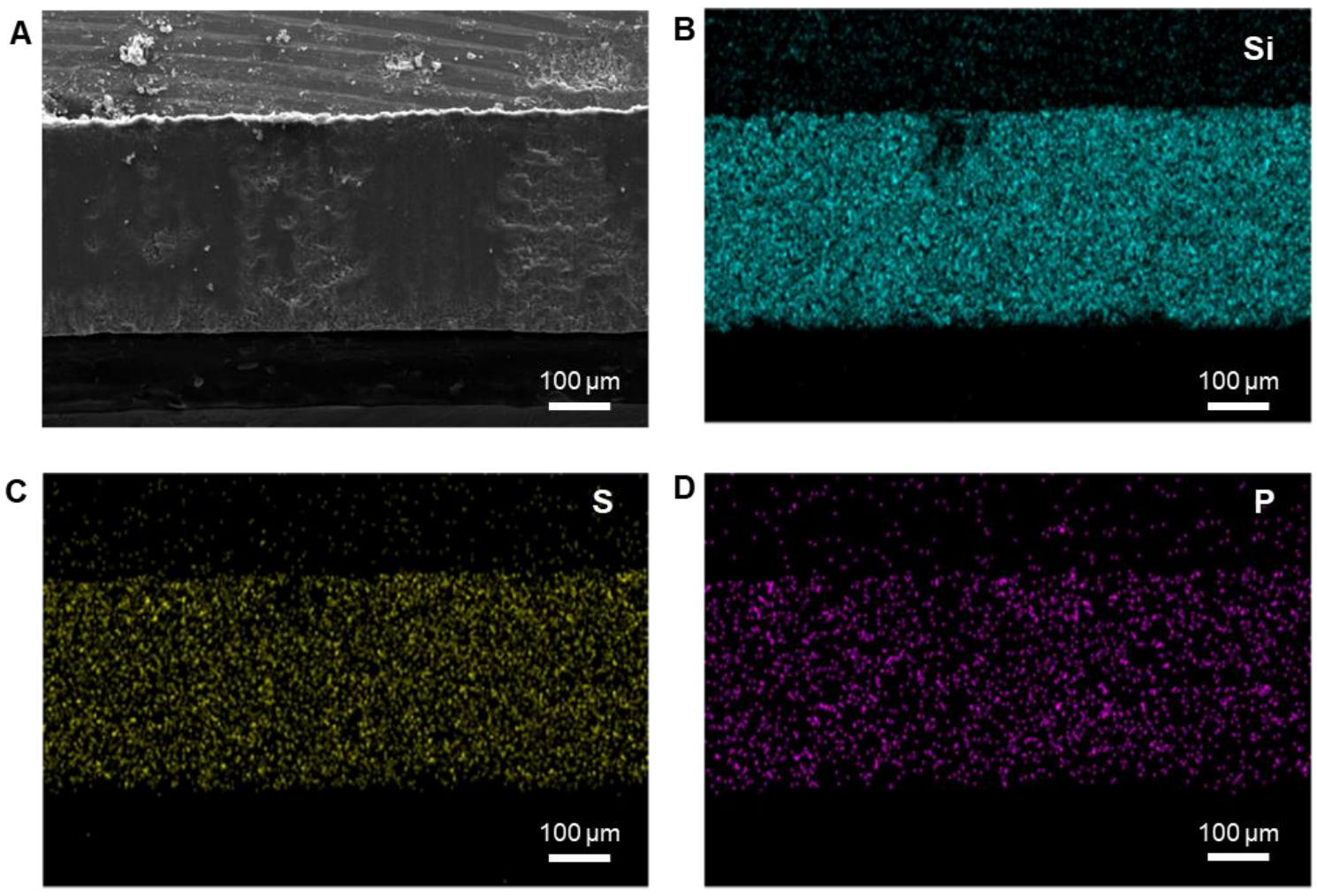


**Fig. S3. Microstructure of the pristine CnSi composite anode.** Cross-sectional SEM images and corresponding energy-dispersive X-ray spectroscopy (EDS) elemental maps of the pristine CnSi composite anode.

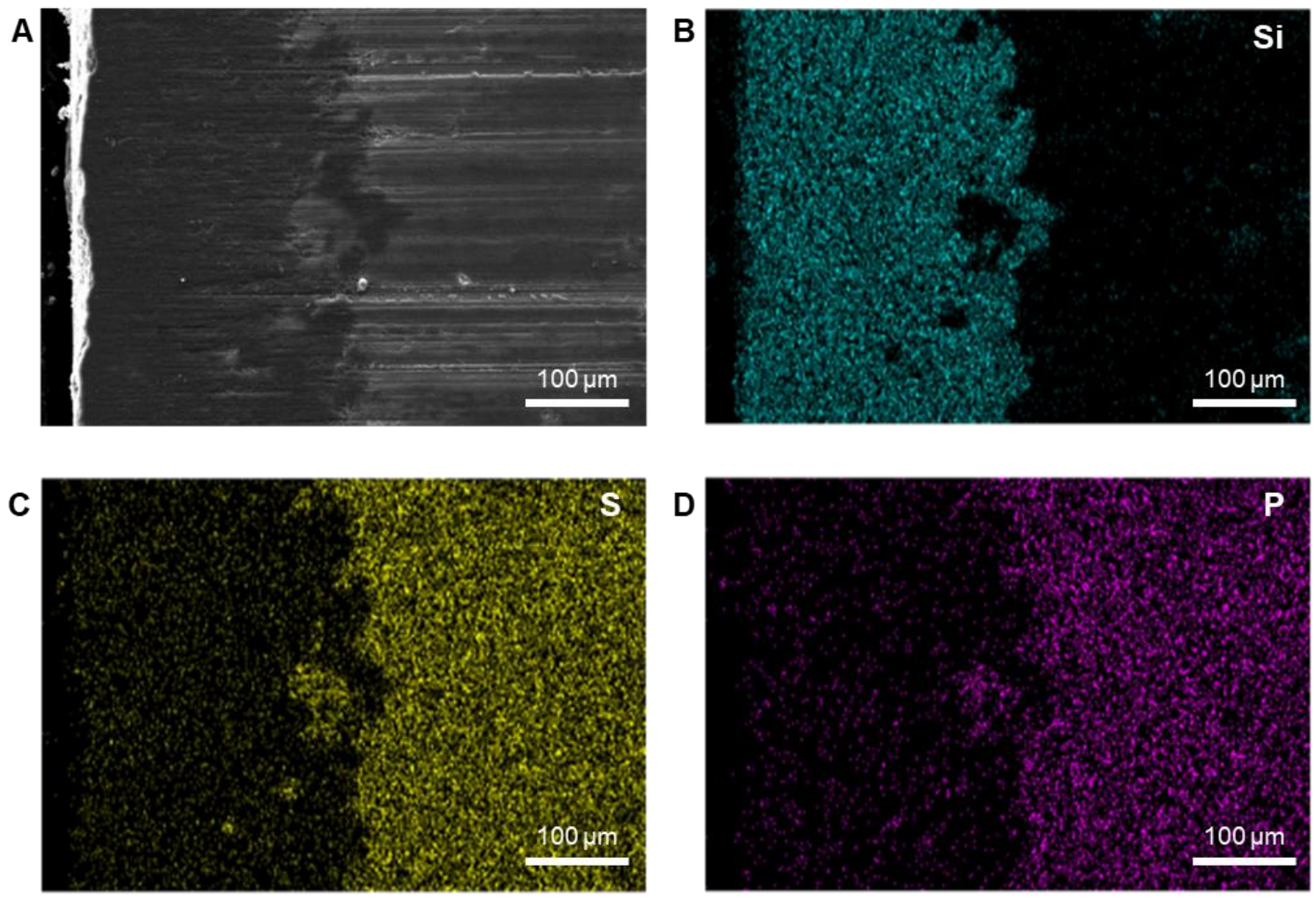


**Fig. S4. Interface between CnSi anode and $Li_6PS_5Cl$ (LPSCl).** Cross-sectional SEM images and EDS elemental maps showing the interface between the CnSi anode and the LPSCl solid electrolyte layer.

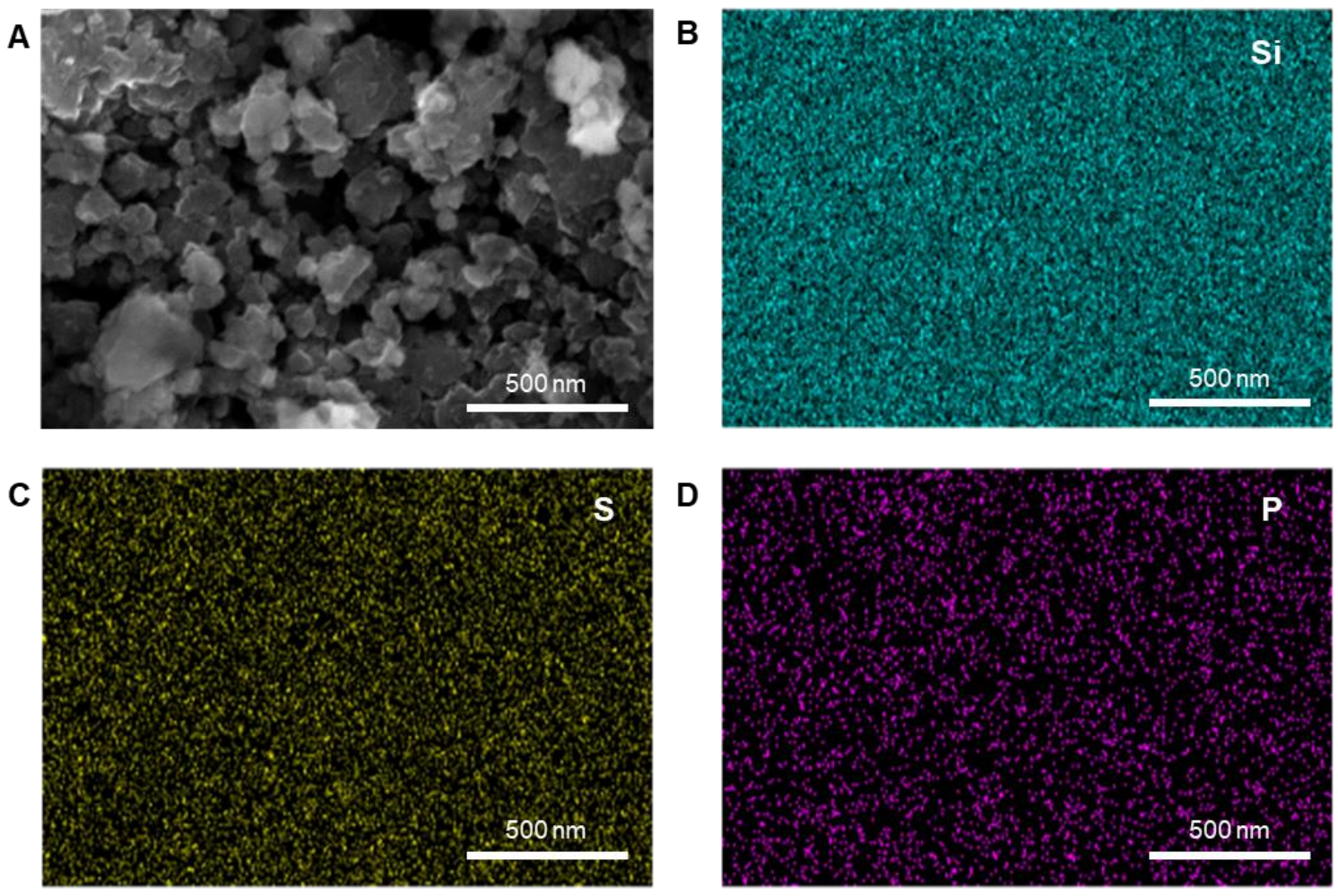


**Fig. S5. Detailed morphology of CnSi composite particles.** High-magnification SEM images and corresponding EDS elemental maps of CnSi composite particles.

**Note S1**: To clarify the microstructure of the composite CnSi anode, cross-sectional scanning electron microscopy (SEM) coupled with energy-dispersive X-ray spectroscopy (EDS) elemental mapping was performed on the pristine electrode. The results are presented in Fig. S3 (overview of the CnSi anode cross-section), Fig. S4 (the CnSi|LPSCl interface region), and Fig. S5 (high-magnification views of composite particles).

The EDS maps in Fig. S3 show that Si, S, and P are uniformly distributed across the entire cross-section of the composite anode, indicating homogeneous mixing of the components. Fig. S4 further demonstrates that this uniformity is maintained at the interface between the CnSi anode and the LPSCl solid electrolyte layer. The high-magnification images in Fig. S5 confirm that within individual composite regions, Si particles, conductive carbon, and LPSCl electrolyte are intimately mixed at the micrometer scale, ensuring effective electronic and ionic percolation pathways.

These microstructural observations validate the successful fabrication of a uniform and well-connected composite anode, which is essential for the reliability of the *in situ* characterization and the reproducibility of the electrochemical performance.

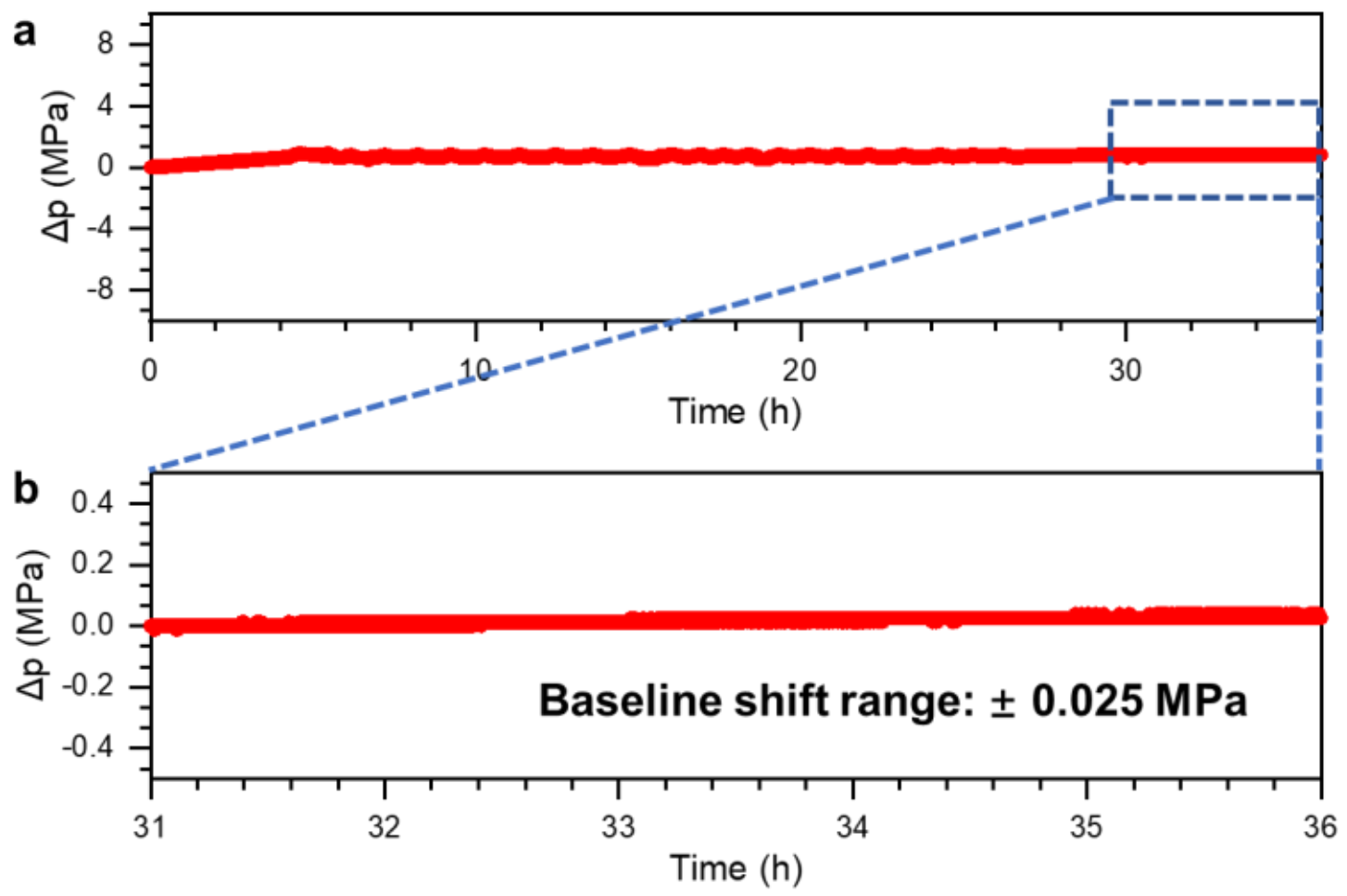


**Fig. S6. Baseline stability assessment for *in situ* stress monitoring.** (a) *In situ* pressure monitoring (PM) curve recorded during a 36-hour rest period under open circuit potential (OCP) prior to electrochemical cycling. (b) Enlarged view of the final 6 hours of the resting period, showing that the baseline pressure fluctuation was confined within ±0.025 MPa, confirming negligible mechanical drift.

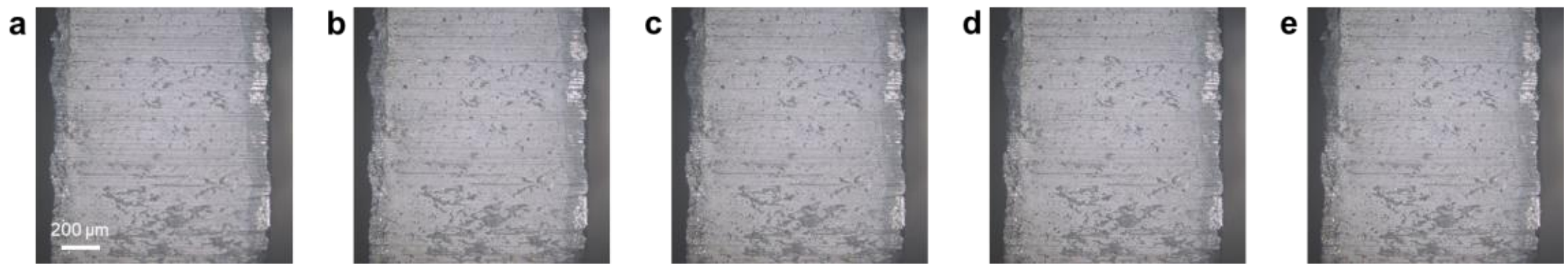


**Fig. S7. Mechanical stability of the all-solid-state battery (ASSB) under open circuit conditions.** (a-e) *In situ* optical microscopy (OM) images of the assembled ASSB acquired during the resting period under open-circuit potential. The images were taken at different time points over the 36-hour stabilization period. No crack formation or morphological changes were observed on either the anode or the cathode side, confirming that the cell structure remained mechanically stable prior to electrochemical cycling.

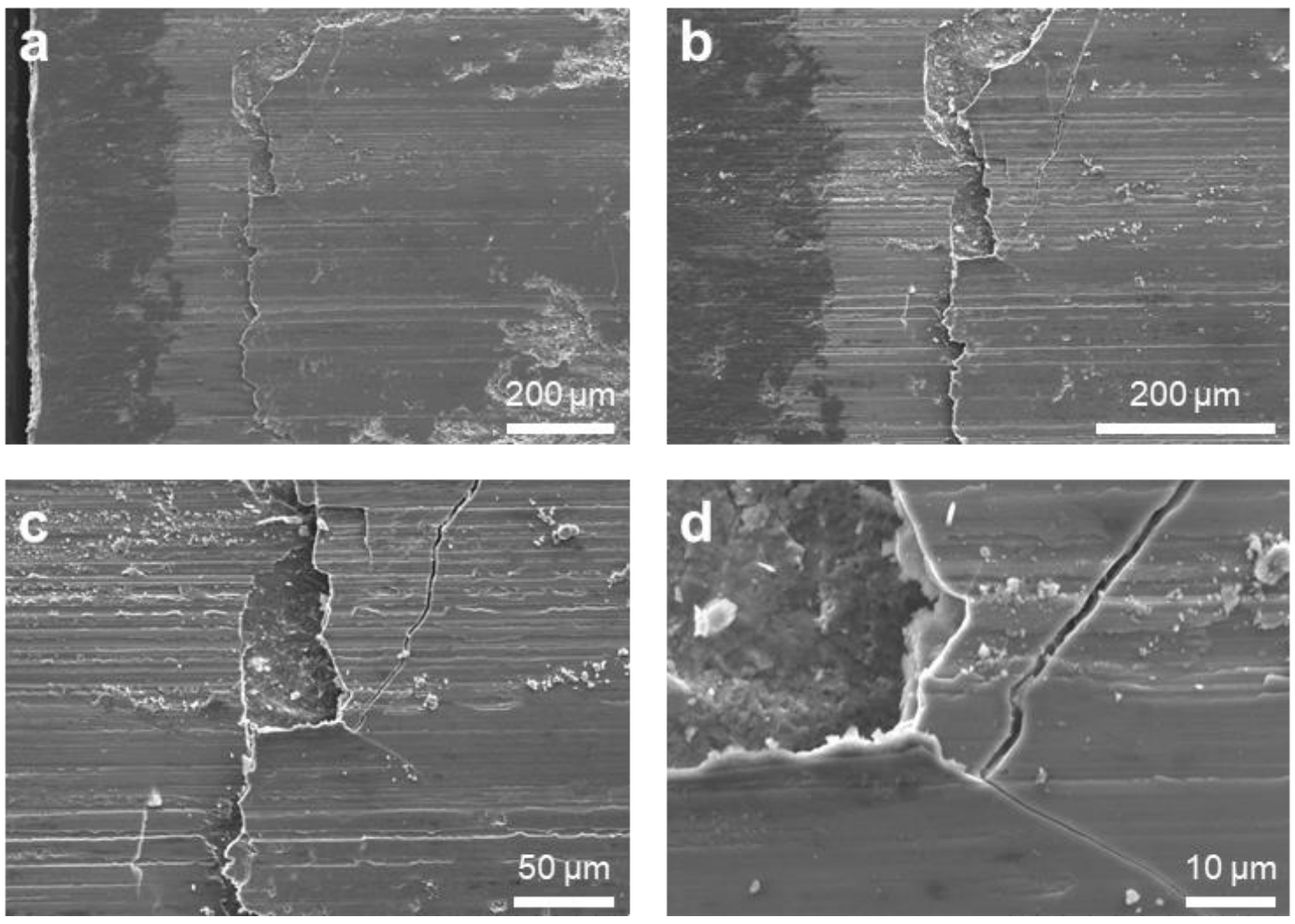


**Fig. S8. Hierarchical crack morphology revealed by *ex situ* SEM.** (a-d) *Ex situ* SEM images of the crack regions after cycling, acquired at increasing magnifications. The images reveal that, in addition to the large cracks observable by OM, numerous smaller branched cracks emanate from the main crack paths, confirming the hierarchical nature of stress-induced mechanical degradation.

**Note S2:** As shown in the progressively magnified views (Fig. S8), the large cracks observed by OM are accompanied by smaller, branched cracks that deviate from the main crack paths. These fine cracks, which are below the resolution limit of OM, become clearly visible at higher magnifications in the SEM images. This hierarchical crack morphology - consisting of both primary macroscopic cracks and secondary microcracks - provides additional microstructural evidence supporting the stress-dominated ECM failure mechanism proposed in our work.

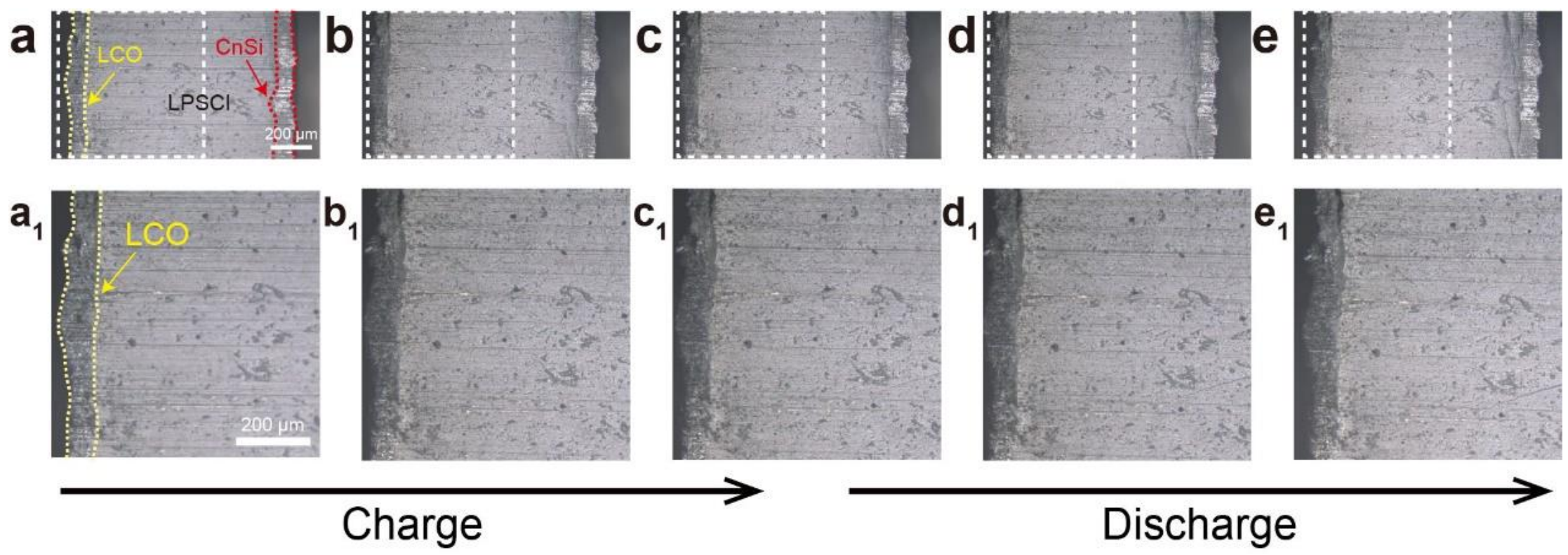


**Fig. S9. *In situ* OM images of the LCO|LPSCl|CnSi ASSB.** *In situ* OM images obtained at OCP (a), during charging (b-c) and during discharging (d-e) of LCO|LPSCl|CnSi ASSB at 0.1C (Same as Fig. 1C-G). Magnified *in situ* OM images for cathode side obtained at OCP ($a_1$), during charging ($b_1$-$c_1$) and during discharging ($d_1$-$e_1$) of LCO|LPSCl|CnSi ASSB at 0.1C.

**Note S3:** Fig. S9($a_1$-$e_1$) present magnified views of the cathode/electrolyte interface corresponding to the regions indicated in Fig. S9(a-e), which is same as Fig. 1C-G. As can be seen from these enlarged images, only rare and minor cracks were observed on the cathode side throughout the entire cycling process. The mechanical failure at the cathode/electrolyte interface was significantly less severe compared to the anode side. This observation is consistent with the intrinsically smaller volume changes and lower stress variations associated with the LCO cathode during lithiation/delithiation, in contrast to the substantial expansion and modulus evolution of the silicon anode.

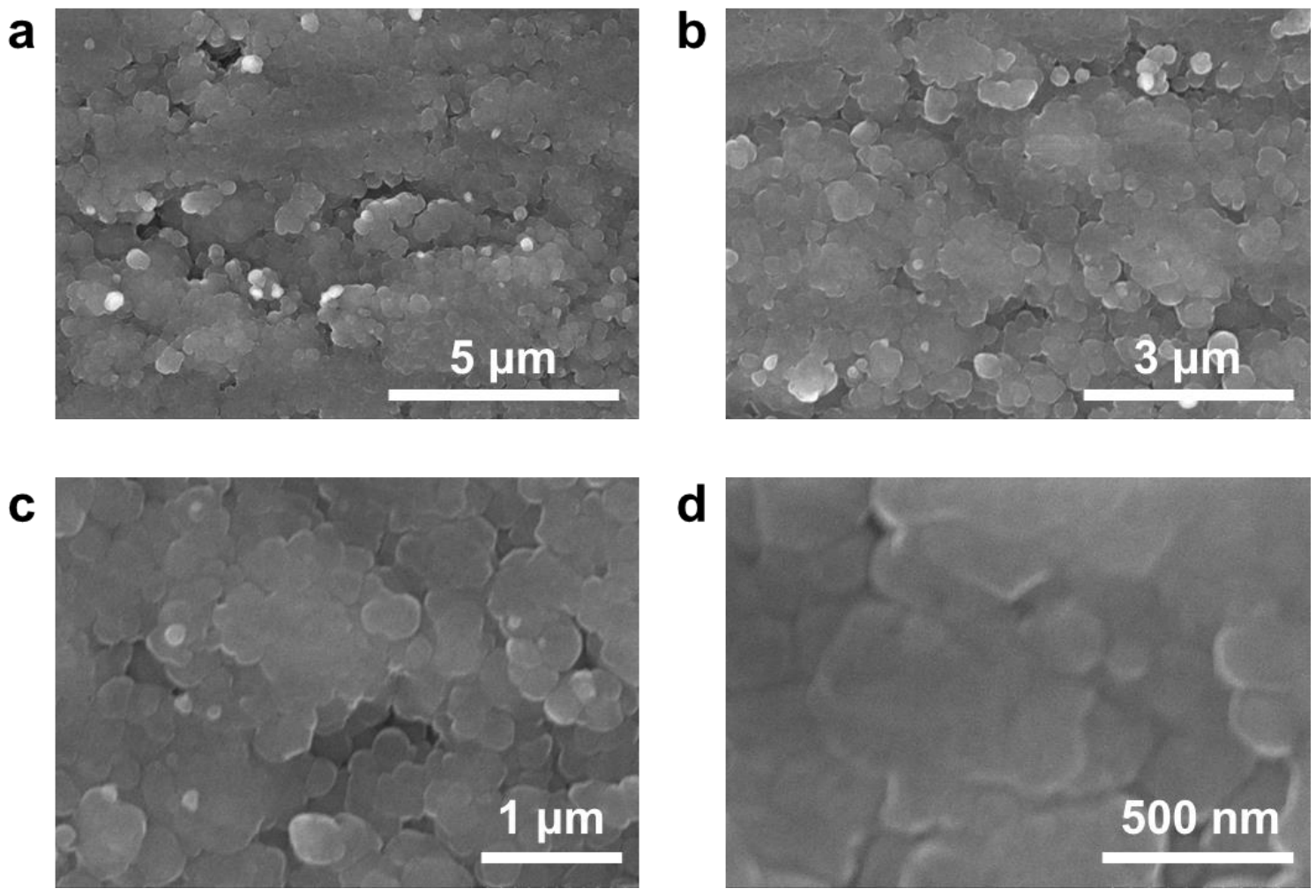


**Fig. S10. Morphology of lithiated CnSi.** Representative SEM images of the CnSi composite anode after lithiation.

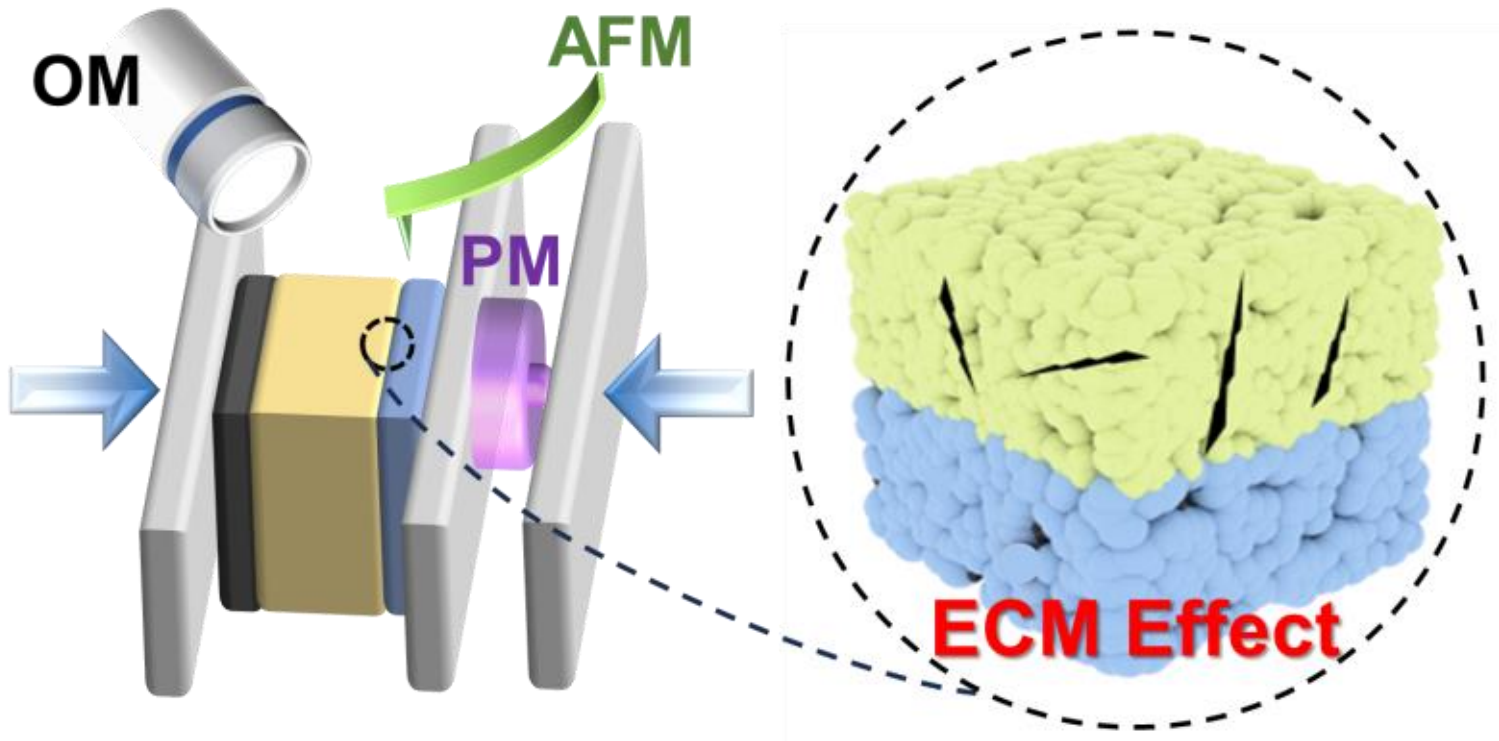


**Fig. S11. A general framework for probing electro-chemo-mechanical (ECM) coupling via *in situ* methodologies.** The suite of *in situ* characterization techniques - including pressure monitoring (PM), optical microscopy (OM), and atomic force microscopy (AFM) - provides a comprehensive platform for decoupling the coupled ECM effects in all-solid-state batteries.

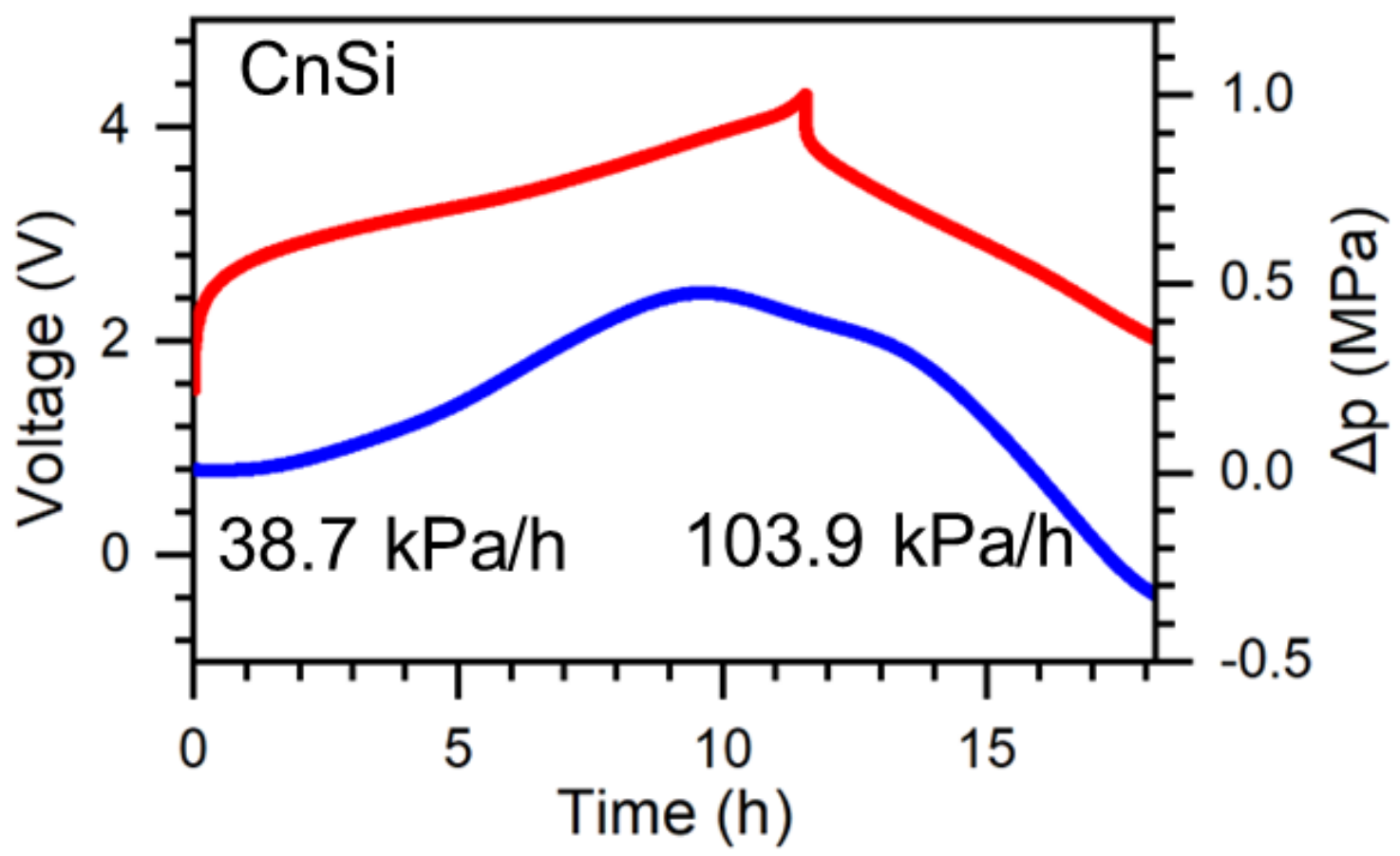


**Fig. S12.** ***In situ*** **PM curves and related charging/discharging curves of the LCO|LPSCl|CnSi ASSB during cycling.**

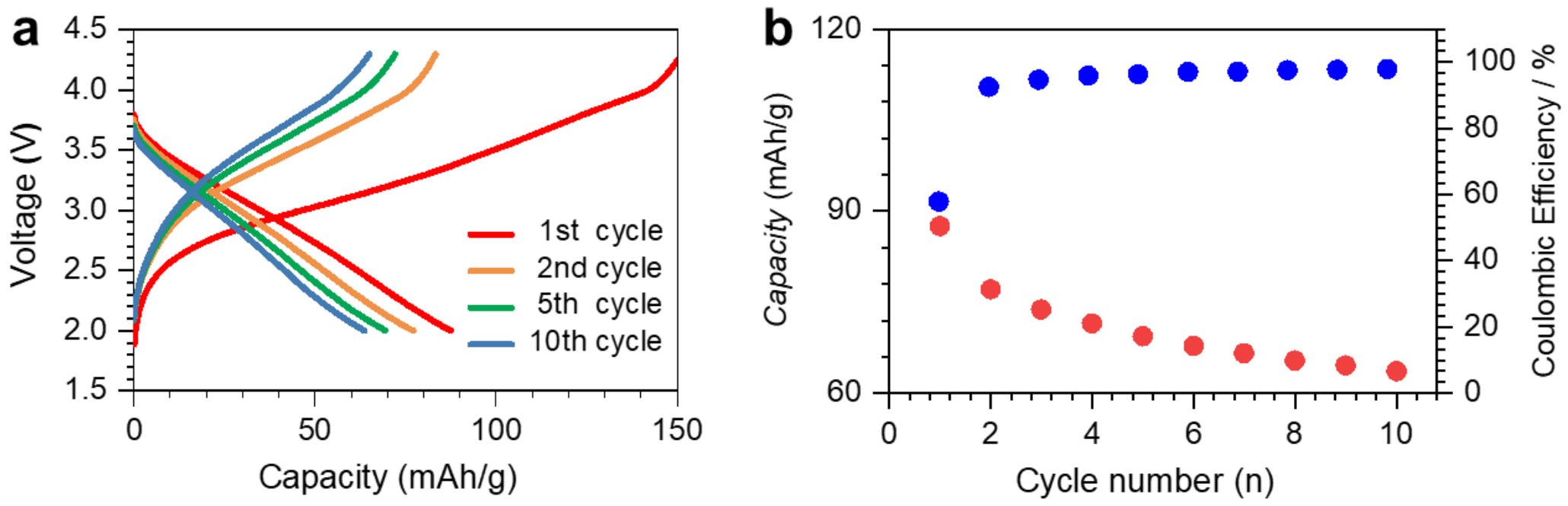


**Fig. S13. Electrochemical performance of CnSi|LPSCl|LCO ASSBs.** Galvanostatic discharge and charge profiles (a) and cycling performance (b) of CnSi|LPSCl|LCO cells for *in situ* PM of different cycles.

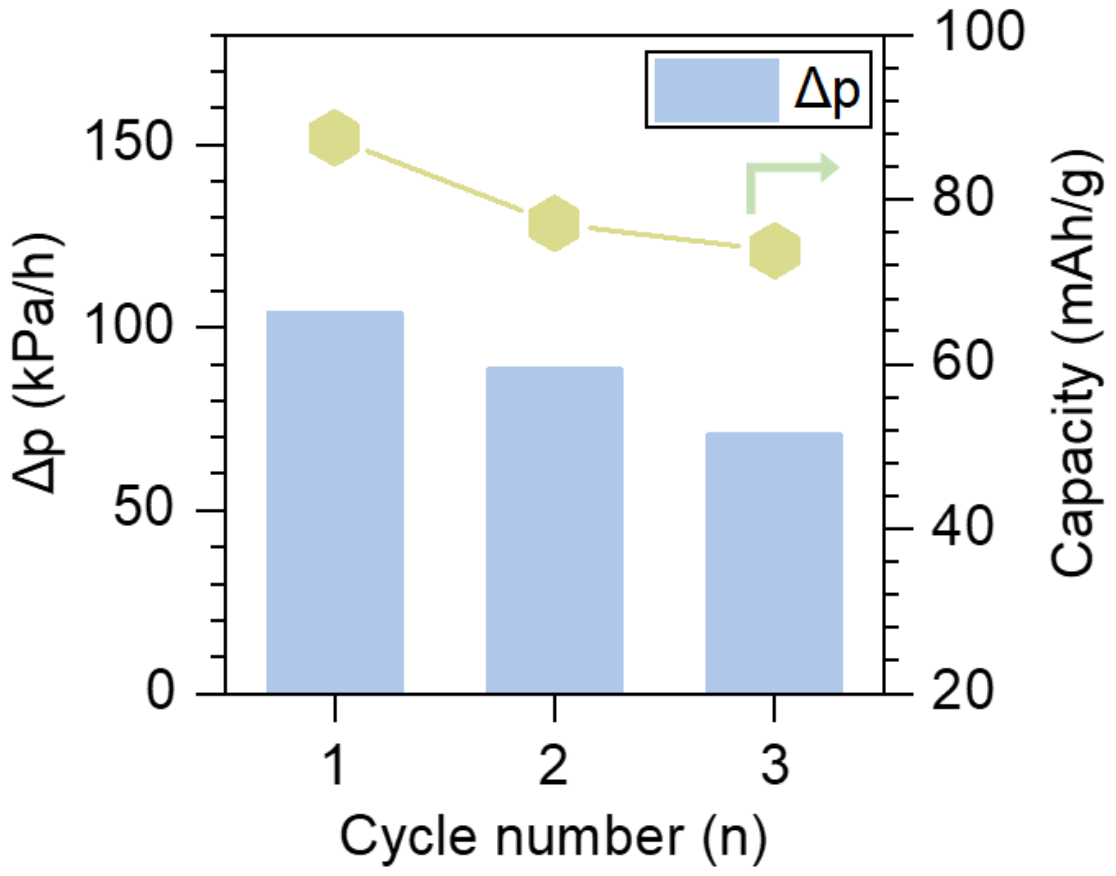


**Fig. S14. Pressure variation and capacity retention of CnSi|LPSCl|LCO cells with cycle number.** The Δp (stress variation) and capacity of CnSi|LPSCl|LCO cells at different cycle numbers.

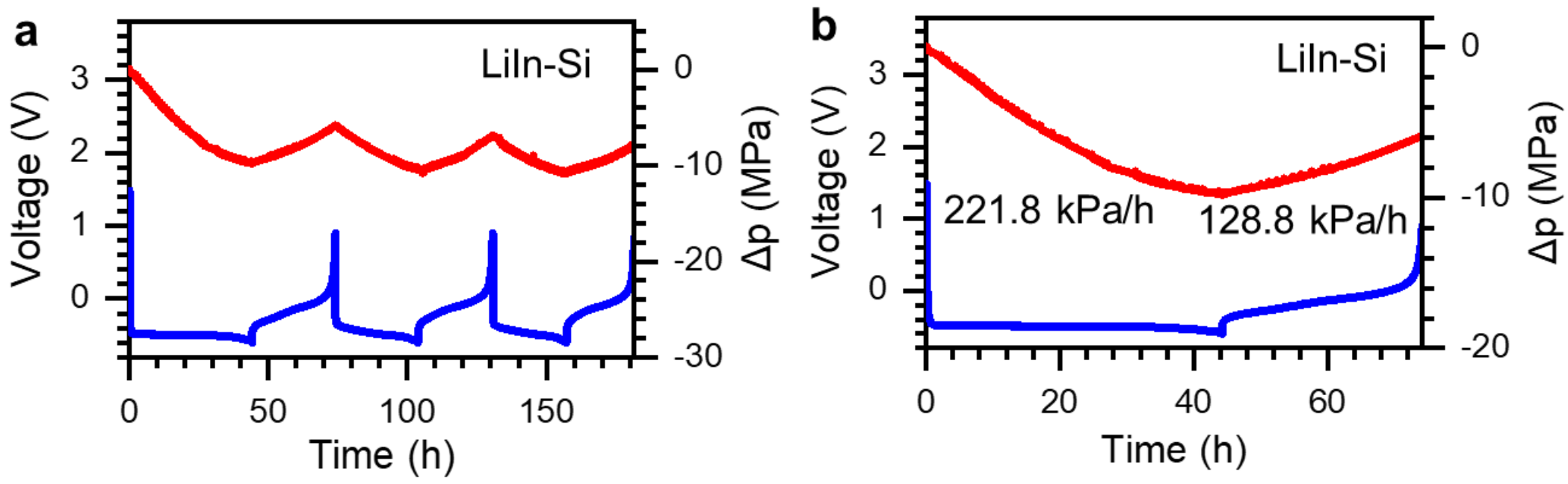


**Fig. S15. *In situ* PM curves and related charging/discharging curves of LiIn-Si ASSB.** (a) *In situ* PM curves and related charging/discharging curves for the first three cycles. (b) *In situ* PM curves and related charging/discharging curves for the first cycle.

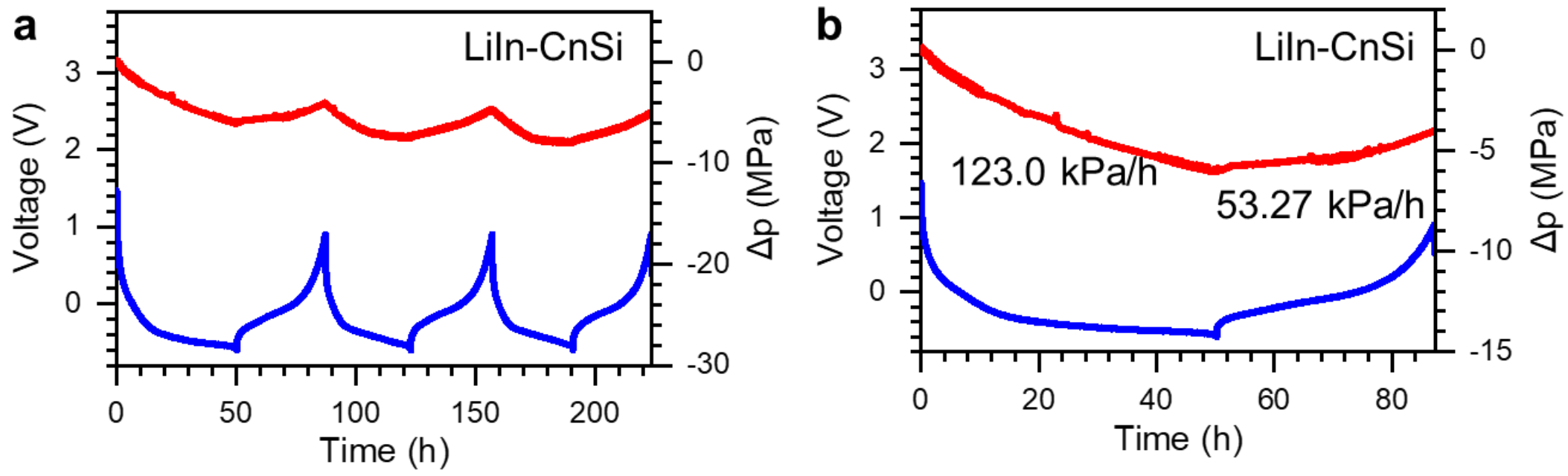


**Fig. S16. *In situ* PM curves and related charging/discharging curves of LiIn-CnSi ASSB.** (a) *In situ* PM curves and related charging/discharging curves for the first three cycles. (b) *In situ* PM curves and related charging/discharging curves for the first cycle.

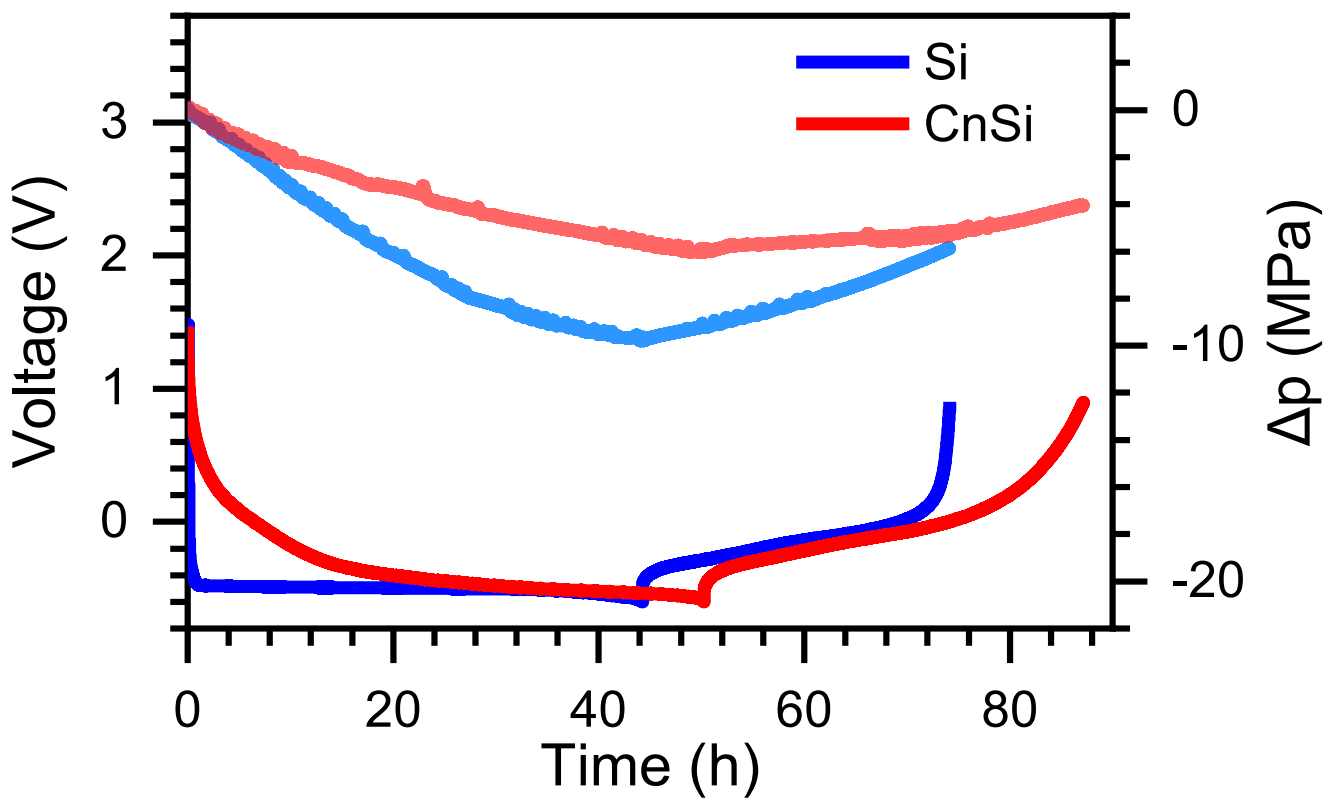


**Fig. S17. Comparison of *in situ* PM between LiIn-Si and LiIn-CnSi ASSBs.** *In situ* PM curves and related charging/discharging curves of LiIn-Si ASSB and LiIn-CnSi ASSB.

**Note S4**: To address the generalizability of the stress-dominated failure mechanism from composite electrodes to Si-only configurations, as well as the potential influence of interfacial chemical degradation, comparative *in situ* PM measurements were performed on two half-cell systems: LiIn-Si (pure Si anode without solid-state electrolyte or carbon additives) and LiIn-CnSi (composite electrode). The corresponding stress evolution curves are presented in Figs. S15 (LiIn-Si) and S16 (LiIn-CnSi), with quantitative stress variation rates compared in Fig. S17 and summarized in Table S3.

As shown in Table S3, for the pure Si anode, the stress variation was 221.8 kPa/h during lithiation and 128.8 kPa/h during delithiation. For the composite CnSi anode, the values were 123.0 kPa/h (lithiation) and 53.27 kPa/h (delithiation). Two important observations emerge:

1. **Stress evolution remains prominent even in the absence of SSE and carbon.** The pure Si anode exhibits even larger stress variations than the composite electrode. This supports the conclusion that the stress-dominated ECM failure mechanism is directly applicable to Si-only systems.

2. **The composite electrode reduces stress variation relative to pure Si,** which is attributed to the presence of the softer solid-state electrolyte (SSE) and carbon phases that partially buffer mechanical mismatch. This does not contradict our mechanistic model but rather illustrates how modulus engineering (lowering the effective modulus of the anode layer) can mitigate stress.

Regarding the concern about interfacial chemical decomposition and interphase formation(*20*), such processes do occur in composite electrodes. However, if chemical degradation were the dominant cause of stress variation, fundamentally different stress evolution patterns would be expected between the pure Si and composite systems. Instead, the stress curves exhibit similar overall shapes (Figs. S15-S17), and the pure Si system, which lacks SSE-induced chemical complexity, shows even higher stress amplitudes. This indicates that mechanical stress from the Si anode remains the primary driver, with chemical contributions playing a secondary role.

Thus, the stress-dominated failure mechanism is general and applicable to both composite and Si-only configurations.

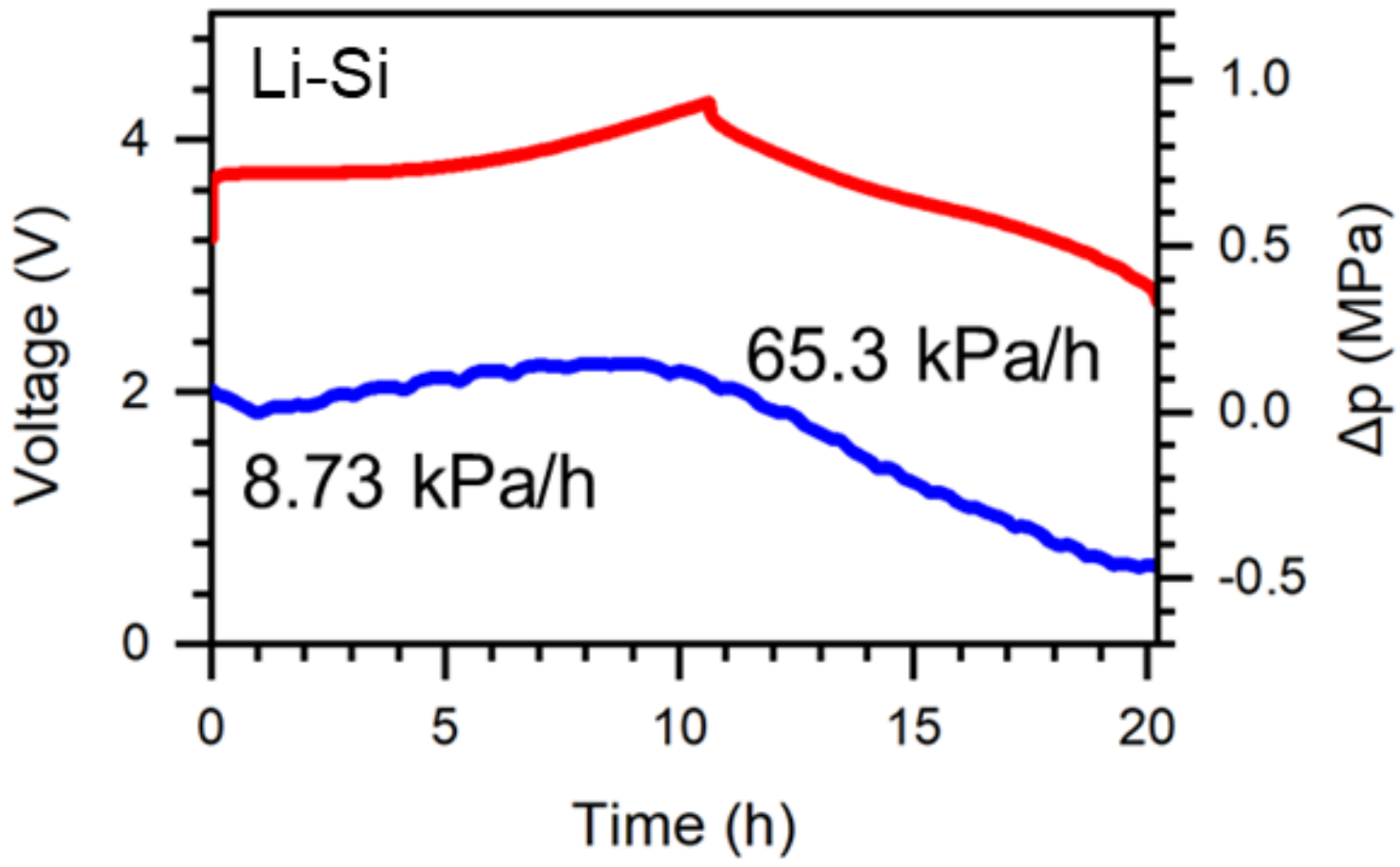


**Fig. S18.** ***In situ*** **PM curves and related charging/discharging curves of LCO|LPSCl|Li-Si ASSB.**

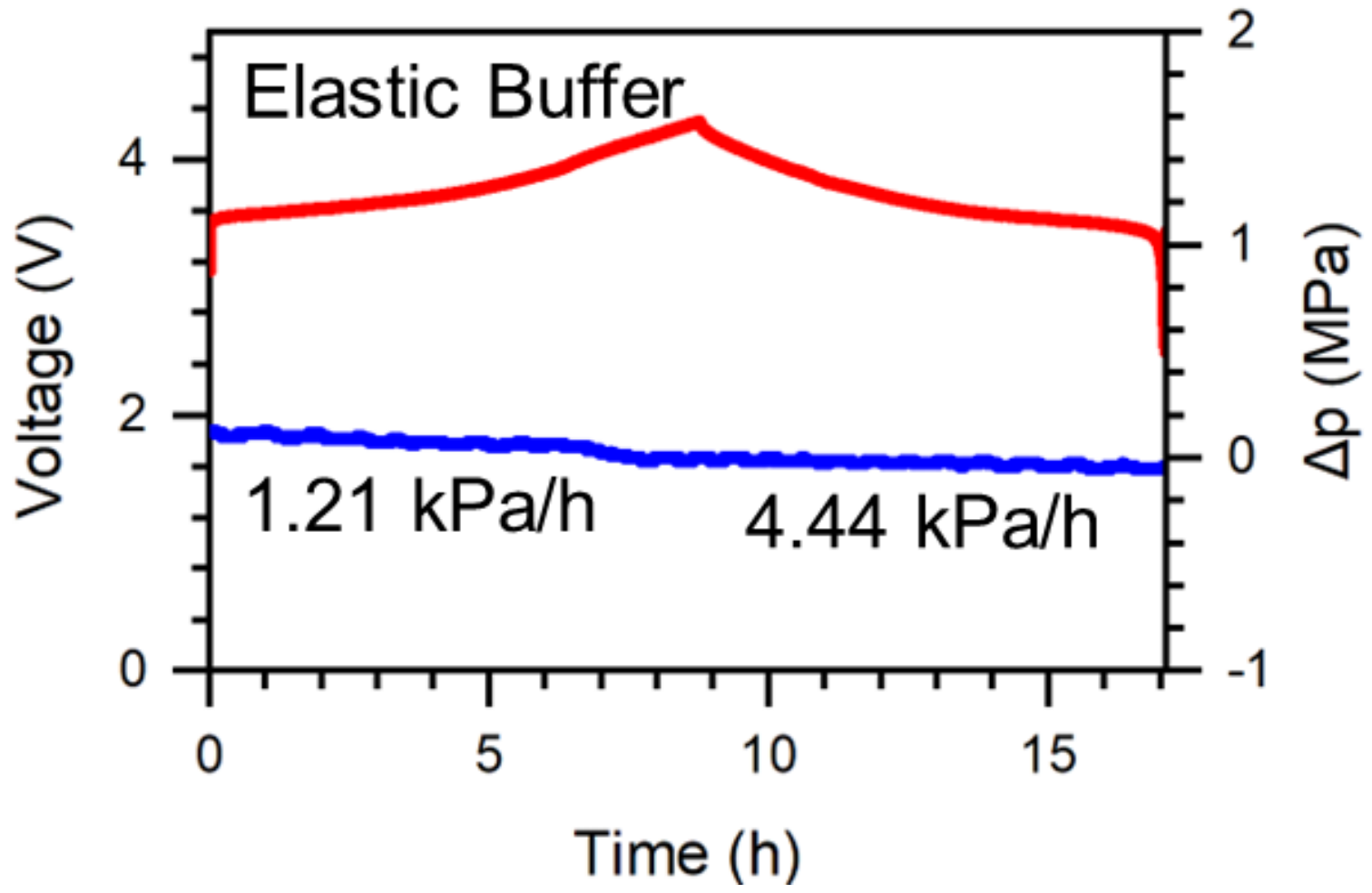


**Fig. S19.** ***In situ*** **PM curves and related charging/discharging curves of LCO|Elastic buffer|Li-Si ASSB.**

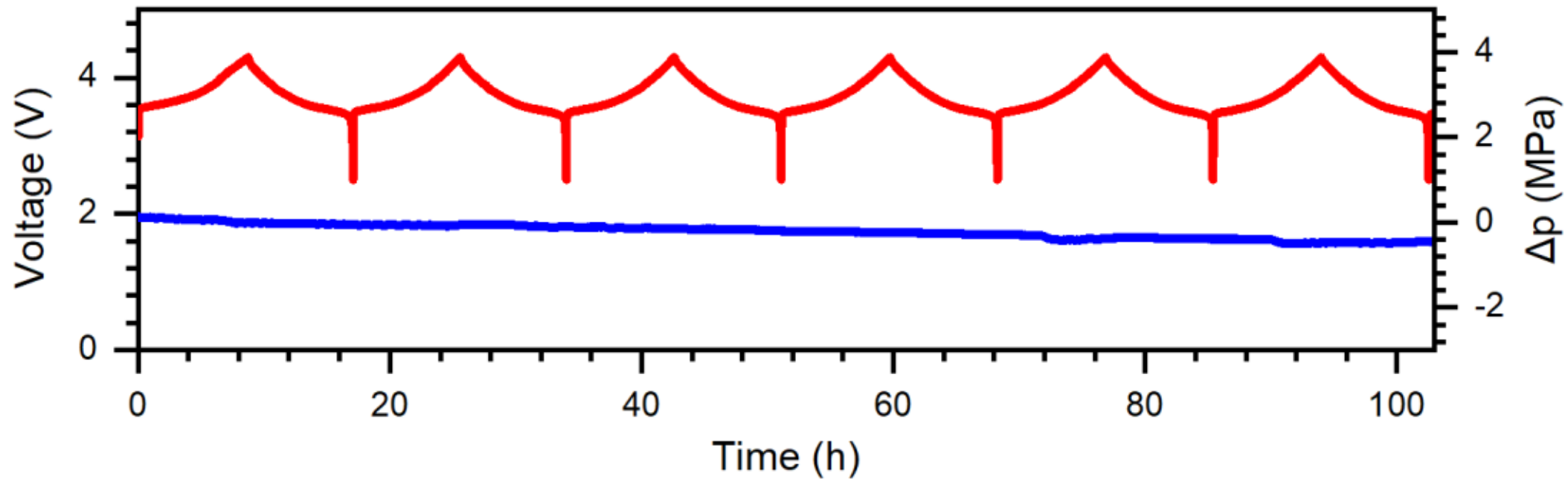


**Fig. S20.** ***In situ*** **PM curves and related charging/discharging curves of LCO|Elastic buffer|Li-Si ASSB for the first six cycles.**

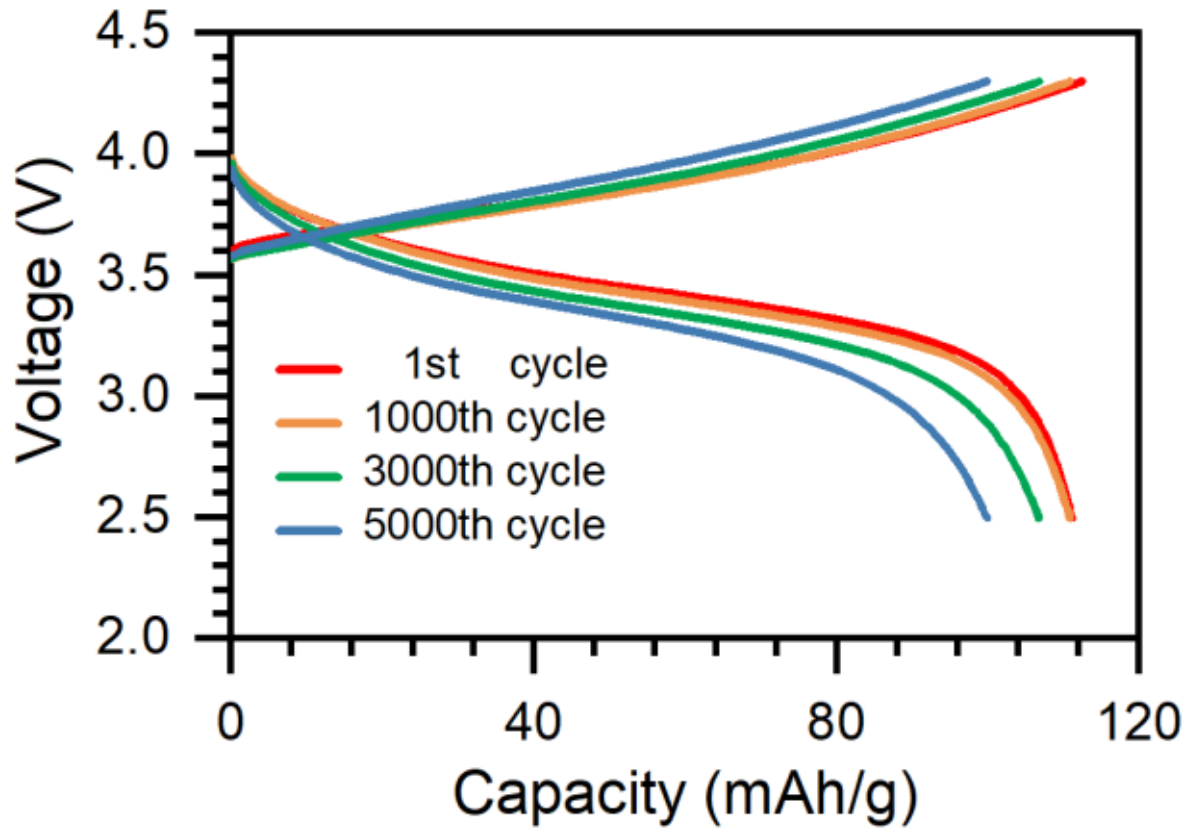


**Fig. S21. Charge and discharge curves of LCO|Elastic buffer|Li-Si ASSBs at 3C.**

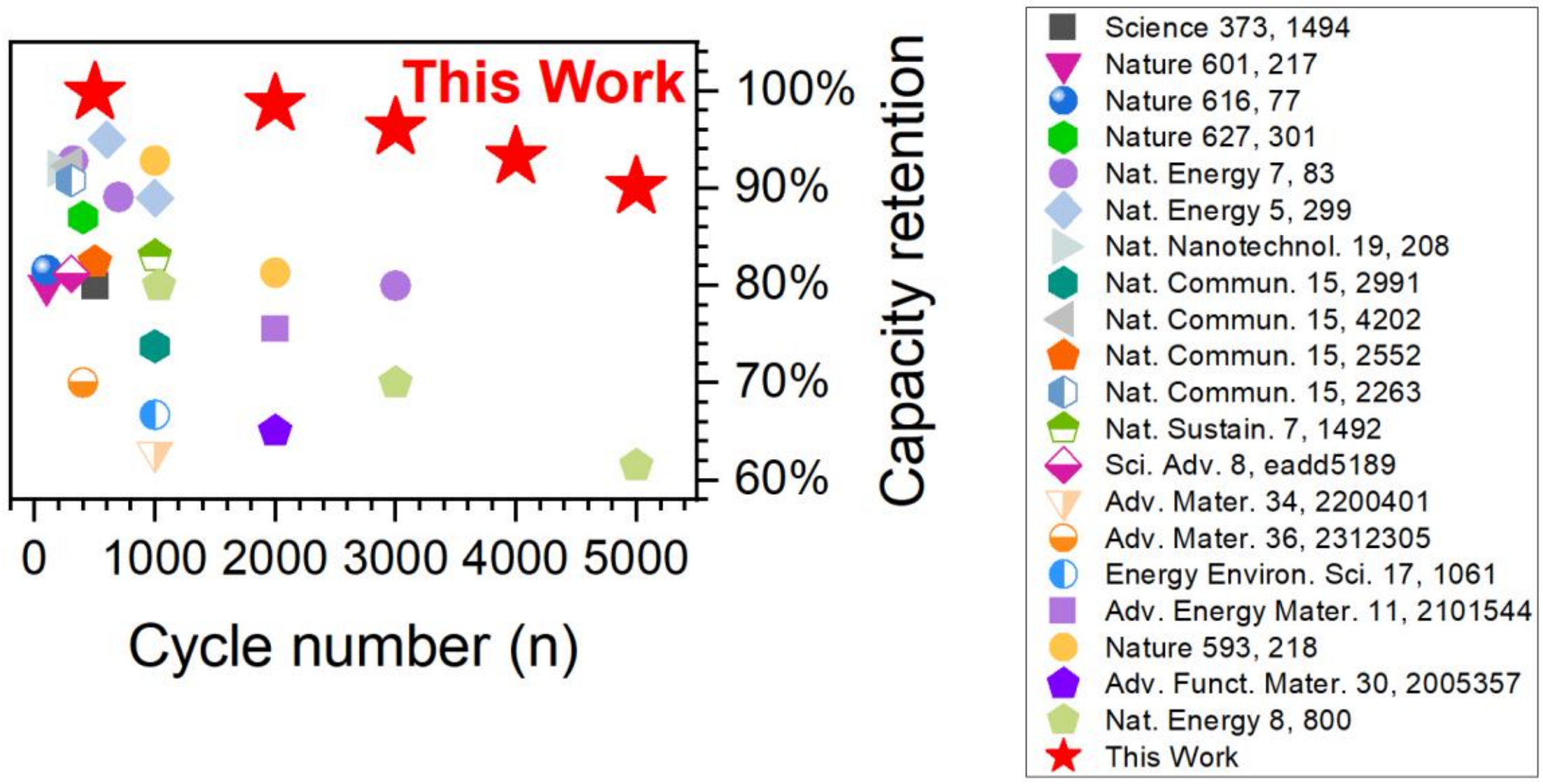


**Fig. S22. Performance comparison with state-of-the-art ASSBs (colored symbols) from peer-reviewed literature.** (*5, 6, 21-38*)

## Supplementary Tables

| | Charge | | | Discharge | |
|---|---|---|---|---|---|
| **State of Charge (SOC)** | **0** | **50%** | **100%** | **50%** | **0** |
| **Si volume ($\times 10^{-3}$ $\mu m^3$)** | 5.11 | 10.45 | 14.03 | 9.03 | 8.48 |
| **Crack area ($\times 10^{3}$ $\mu m^2$)** | 0 | 5.58 | 7.37 | 12.57 | 16.63 |

**Table S1. The Si volume and crack area at different state of charge (SOC).**

| **Cycle number (n)** | **1** | **2** | **3** |
|---|---|---|---|
| **Δp (kPa/h)** | 103.99 | 88.5 | 70.6 |
| **Capacity (mAh/g)** | 87.5 | 77.1 | 73.8 |

**Table S2. The Δp and capacity of CnSi|LPSCl|LCO cells at different cycle numbers.**

| | Stress Variation (kPa/h) | |
|---|---|---|
| | **Charge** | **Discharge** |
| **Si** | 221.8 | 128.8 |
| **CnSi** | 123.0 | 53.27 |

**Table S3. Stress variation for ASSBs with Si and CnSi.**

| | Stress Variation (kPa/h) | |
|---|---|---|
| | **Charge** | **Discharge** |
| **CnSi** | 38.7 | 103.9 |
| **Li-Si** | 8.73 | 65.3 |
| **Elastic Buffer Layer** | 1.21 | 4.44 |

**Table S4. Quantitative summary of stress variation during charging and discharging for ASSBs with CnSi, Li-Si and elastic buffer layer.**

| Cycling number (n) | Capacity retention | Temperature | References |
|---|---|---|---|
| 500 | 80% | RT | *Science* 373, 1494 (Ref. (*5*)) |
| 100 | 80% | 30 $^{o}$C | *Nature* 601, 217 (Ref. (*25*)) |
| 100 | 81.6% | 30 $^{o}$C | *Nature* 616, 77 (Ref. (*28*)) |
| 400 | 87% | 25 $^{o}$C | *Nature* 627, 301 (Ref. (*37*)) |
| 1000 | 92.8% | 55 $^{o}$C | *Nature* 593, 218 (Ref. (*24*)) |
| 2000 | 81.3% | | |
| 320 | 92.8% | RT | *Nat. Energy* 7, 83 (Ref. (*27*)) |
| 695 | 89.1% | | |
| 3000 | 80% | | |
| 600 | 95% | 60 $^{o}$C | *Nat. Energy* 5, 299 (Ref. (*22*)) |
| 1000 | 89% | | |
| 1033 | 80% | 55 $^{o}$C | *Nat. Energy* 8, 800 (Ref. (*38*)) |
| 3000 | 70% | | |
| 5000 | 61.5% | | |
| 200 | 92% | 35 $^{o}$C | *Nat. Nanotechnol.* 19, 208 (Ref. (*35*)) |
| 1000 | 83% | RT | *Nat. Sustain.* 7, 1492 (Ref. (*31*)) |
| 1000 | 73.8% | RT | *Nat. Commun.* 15, 2991 (Ref. (*29*)) |

RT: Room Temperature

**Table S5. Performance comparison with state-of-the-art ASSBs from peer-reviewed literature.**

| Cycling number (n) | Capacity retention | Temperature | References |
|---|---|---|---|
| 300 | 92.3% | 30 ºC | *Nat. Commun.* 15, 4202 (Ref. (*33*)) |
| 500 | 82.4% | 25 ºC | *Nat. Commun.* 15, 2552 (Ref (*34*)) |
| 300 | 90.8% | 25 ºC | *Nat. Commun.* 15, 2263 (Ref. (*32*)) |
| 300 | 81.2% | RT | *Sci. Adv.* 8, eadd5189 (Ref. (*26*)) |
| 1000 | 62.9% | RT | *Adv. Mater.* 34, 2200401 (Ref. (*6*)) |
| 400 | 70.03% | 25 ºC | *Adv. Mater.* 36, 2312305 (Ref. (*30*)) |
| 1000 | 66.7% | 55 ºC | *Energy Environ. Sci.* 17, 1061 (Ref. (*36*)) |
| 2000 | 65% | 60 ºC | *Adv. Funct. Mater.* 30, 2005357 (Ref. (*21*)) |
| 1000 | 48% | - | *Adv. Energy Mater.* 11, 2101544 (Ref. (*23*)) |
| 2000 | 75.6% | | |
| 1000 | 84.4% | 30 ºC | *Small* 21, 2411451 (Ref. (*42*)) |
| 2000 | 73.1% | | |
| 500 | 99.8% | RT | This work |
| 2000 | 98.6% | | |
| 3000 | 96.2% | | |
| 4000 | 93.2% | | |
| 5000 | 90.1% | | |

RT: Room Temperature

**Table S5-continued. Performance comparison with state-of-the-art ASSBs from peer-reviewed literature.** (*5, 6, 21-38, 42*)

# Supplementary Discussions

## Supplementary Discussion 1: Phase-dependent modulus of Si-based anodes

Phase analysis of Li-Si alloy anodes reveals the formation of distinct phases including LiSi, $Li_{12}Si_7$, $Li_7Si_3$, $Li_{13}Si_4$, and $Li_{15}Si_4$ during electrochemical cycling, consistent with the Li-Si phase diagram. Through density functional theory (DFT) calculations, we systematically determined the fundamental mechanical properties of these phases, including bulk modulus (B), shear modulus (G), Young's modulus (E), and Poisson's ratio (ν). These parameters were derived through the Voigt-Reuss-Hill approximation of the calculated elastic constants. For comprehensive analysis of the anisotropic mechanical behavior, we employed the ELATE platform to visualize the full elastic tensors and quantify directional variations in mechanical response. Notably, the results demonstrate a progressive mechanical softening with increasing Li content, with $Li_{15}Si_4$ exhibiting a 65.6% reduction in the Young's modulus (54.25 GPa) compared to pure Si (157.59 GPa).

For Si, the calculated stiffness matrix and mechanical properties were shown in Table S6 and Fig. S23. The average mechanical properties of Si calculated by the Voigt-Reuss-Hill approximation were summarized in Table S7, with the calculated Young's modulus by the Hill approximation to be 157.59 GPa.

For LiSi, the calculated stiffness matrix and mechanical properties were shown in Table S8 and Fig. S24. The average mechanical properties of LiSi calculated by the Voigt-Reuss-Hill approximation were summarized in Table S9, with the calculated Young's modulus by the Hill approximation to be 91.61 GPa.

For $Li_{12}Si_7$, the calculated stiffness matrix and mechanical properties were shown in Table S10 and Fig. S25. The average mechanical properties of $Li_{12}Si_7$ calculated by the Voigt-Reuss-Hill approximation were summarized in Table S11, with the calculated Young's modulus by the Hill approximation to be 73.82 GPa.

For $Li_7Si_3$, the calculated stiffness matrix and mechanical properties were shown in Table S12 and Fig. S26. The average mechanical properties of $Li_7Si_3$ calculated by the Voigt-Reuss-Hill approximation were summarized in Table S13, with the calculated Young's modulus by the Hill approximation to be 71.94 GPa.

For $Li_{13}Si_4$, the calculated stiffness matrix and mechanical properties were shown in Table S14 and Fig. S27. The average mechanical properties of $Li_{13}Si_4$ calculated by the Voigt-Reuss-Hill approximation were summarized in Table S15, with the calculated Young's modulus by the Hill approximation to be 67.14 GPa.

For $Li_{15}Si_4$, the calculated stiffness matrix and mechanical properties were shown in Table S16 and Fig. S28. The average mechanical properties of $Li_{15}Si_4$ calculated by the Voigt-Reuss-Hill approximation were summarized in Table S17, with the calculated Young's modulus by the Hill approximation to be 54.25 GPa.

| | | | | | |
|---|---|---|---|---|---|
| 160.16 | 61.73 | 61.73 | 0 | 0 | 0 |
| 61.73 | 160.16 | 61.73 | 0 | 0 | 0 |
| 61.73 | 61.73 | 160.16 | 0 | 0 | 0 |
| 0 | 0 | 0 | 77.24 | 0 | 0 |
| 0 | 0 | 0 | 0 | 77.24 | 0 |
| 0 | 0 | 0 | 0 | 0 | 77.24 |

**Table S6. The calculated stiffness matrix (coefficients in GPa) of Si.**

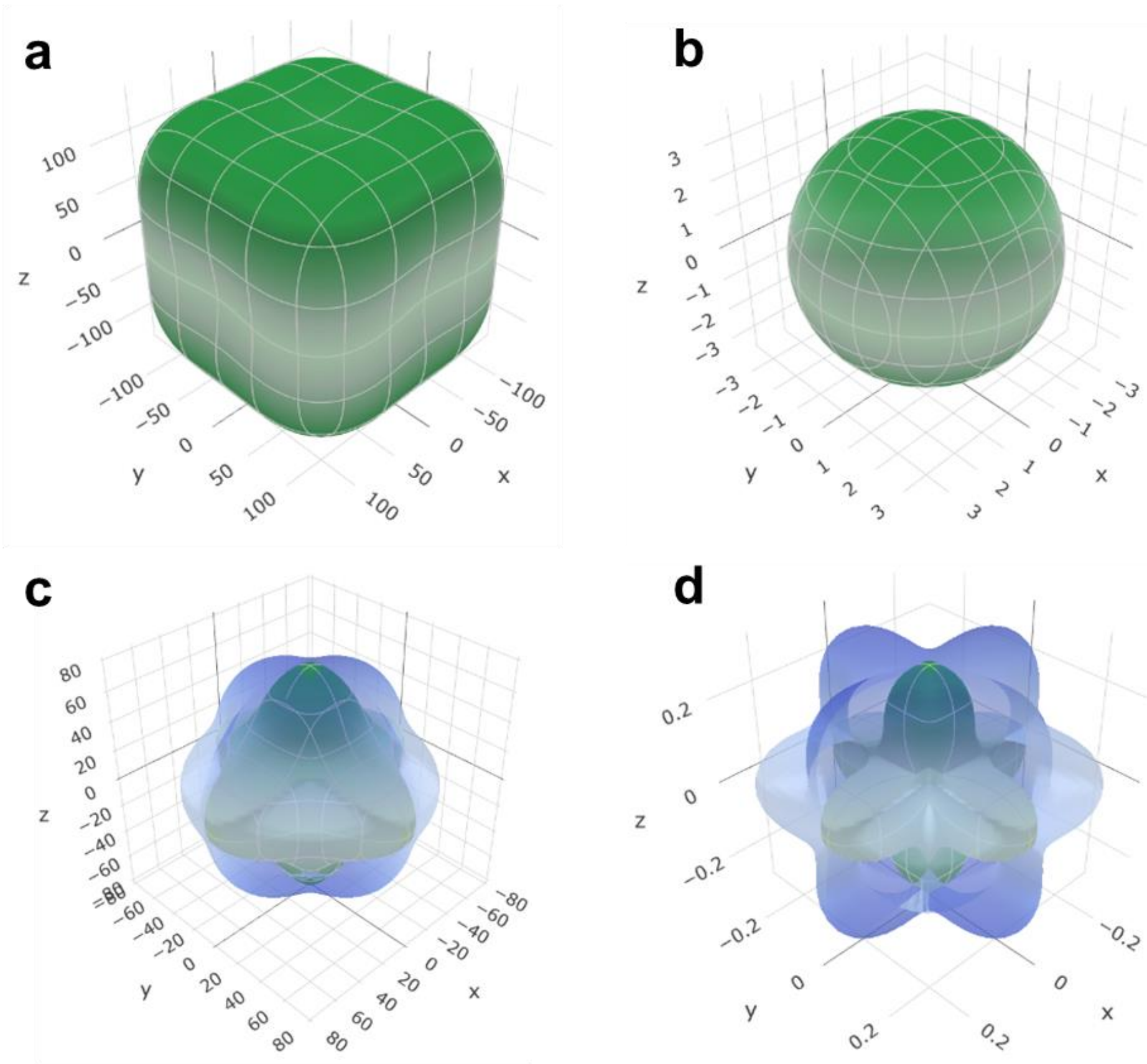


**Fig. S23. Calculated mechanical properties of Si.** (a) Spatial dependence of the Young's modulus. (b) Spatial dependence of linear compressibility. (c) Spatial dependence of the shear modulus. (d) Spatial dependence of the Poisson's ratio.

| Averaging scheme | Bulk modulus (GPa) | Young's modulus (GPa) | Shear modulus (GPa) | Poisson's ratio |
|---|---|---|---|---|
| Voigt | 94.54 | 160.68 | 66.03 | 0.22 |
| Reuss | 94.54 | 154.47 | 62.91 | 0.23 |
| Hill | 94.54 | 157.59 | 64.47 | 0.22 |

**Table S7. The average mechanical properties of Si calculated by the Voigt-Reuss-Hill approximation.**

| | | | | | |
|---|---|---|---|---|---|
| 80.61 | 39.45 | 39.40 | -0.30 | 0.21 | -0.15 |
| 39.45 | 101.90 | 26.23 | 0.06 | -0.22 | -0.09 |
| 39.40 | 26.23 | 102.07 | -0.11 | 0.48 | -0.09 |
| -0.30 | 0.06 | -0.11 | 46.72 | -0.11 | 0.09 |
| 0.21 | -0.22 | 0.48 | -0.11 | 41.70 | -0.07 |
| -0.15 | -0.09 | -0.09 | 0.09 | -0.07 | 46.60 |

**Table S8. The calculated stiffness matrix (coefficients in GPa) of LiSi.**

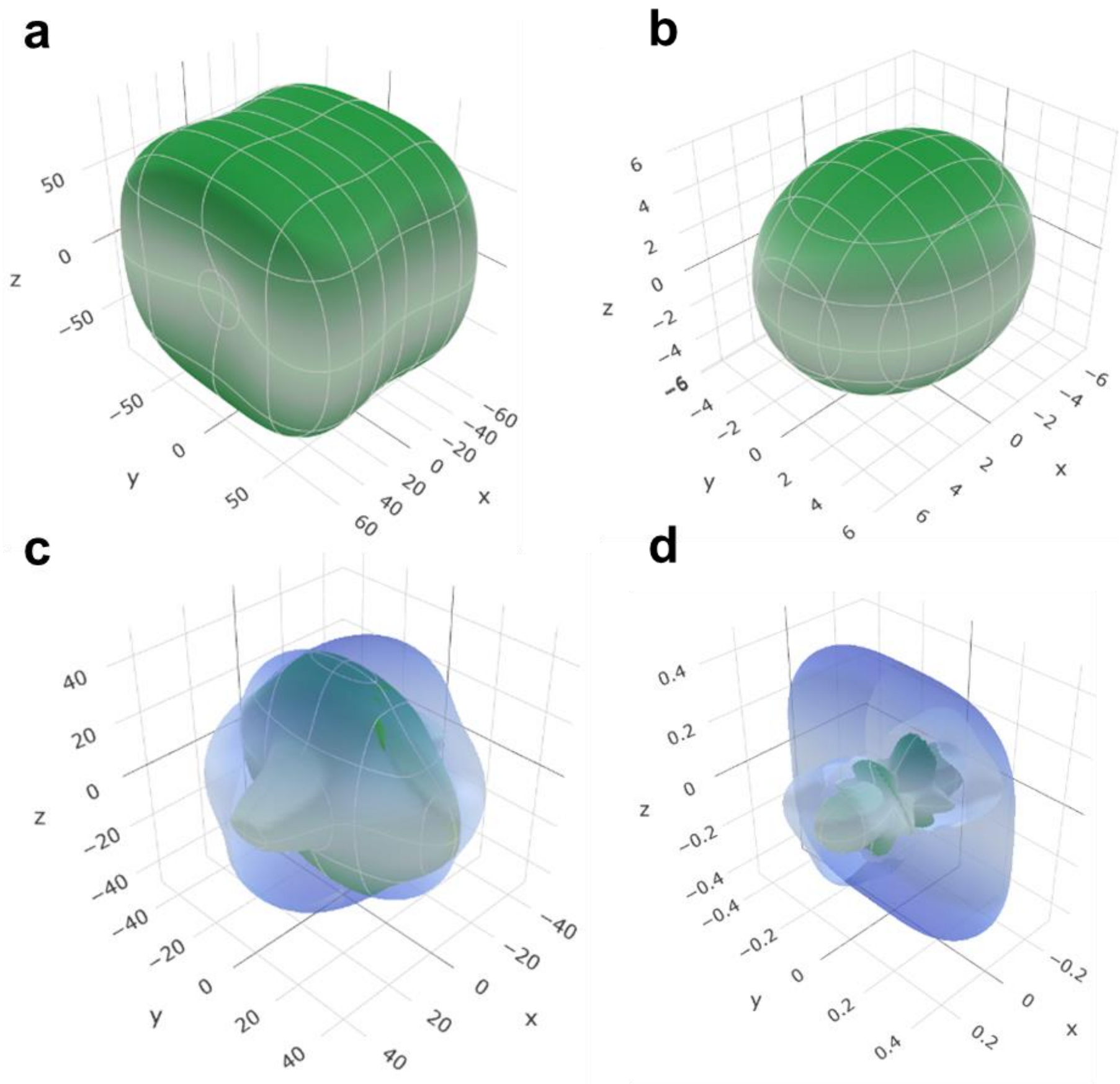


**Fig. S24. Calculated mechanical properties of LiSi.** (a) Spatial dependence of the Young's modulus. (b) Spatial dependence of linear compressibility. (c) Spatial dependence of the shear modulus. (d) Spatial dependence of the Poisson's ratio.

| Averaging scheme | Bulk modulus (GPa) | Young's modulus (GPa) | Shear modulus (GPa) | Poisson's ratio |
|---|---|---|---|---|
| Voigt | 54.97 | 94.57 | 38.97 | 0.21 |
| Reuss | 54.86 | 88.61 | 36.00 | 0.23 |
| Hill | 54.92 | 91.61 | 37.49 | 0.22 |

**Table S9. The average mechanical properties of LiSi calculated by the Voigt-Reuss-Hill approximation.**

| | | | | | |
|---|---|---|---|---|---|
| 90.48 | 10.18 | 5.48 | 0 | 0 | 0 |
| 10.18 | 91.23 | 9.15 | 0 | 0 | 0 |
| 5.48 | 9.15 | 96.58 | 0 | 0 | 0 |
| 0 | 0 | 0 | 26.81 | 0 | 0 |
| 0 | 0 | 0 | 0 | 27.55 | 0 |
| 0 | 0 | 0 | 0 | 0 | 24.43 |

**Table S10. The calculated stiffness matrix (coefficients in GPa) of $Li_{12}Si_7$.**

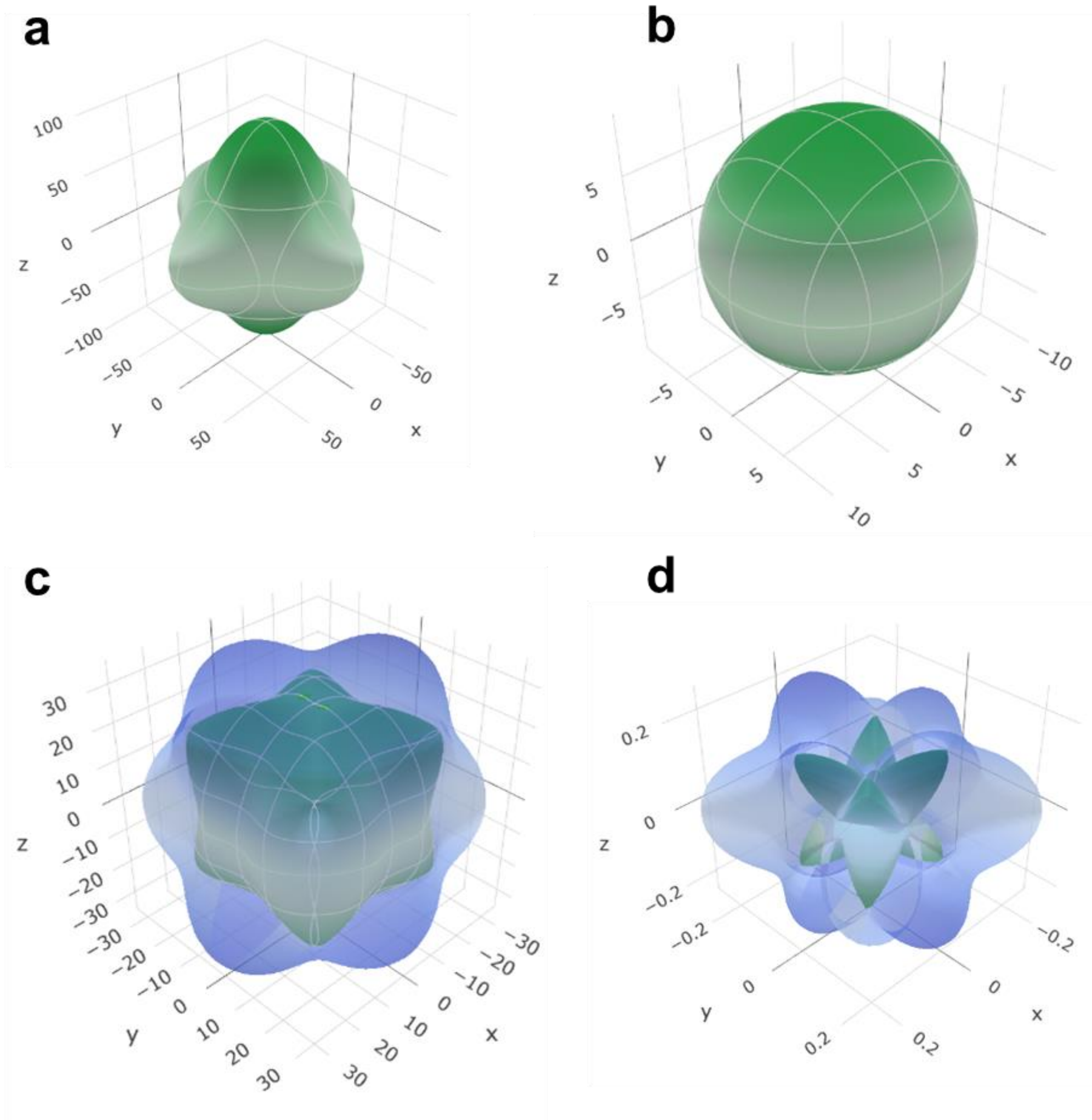


**Fig. S25. Calculated mechanical properties of $Li_{12}Si_7$.** (a) Spatial dependence of the Young's modulus. (b) Spatial dependence of linear compressibility. (c) Spatial dependence of the shear modulus. (d) Spatial dependence of the Poisson's ratio.

| Averaging scheme | Bulk modulus (GPa) | Young's modulus (GPa) | Shear modulus (GPa) | Poisson's ratio |
|---|---|---|---|---|
| Voigt | 36.43 | 75.43 | 32.66 | 0.15 |
| Reuss | 36.41 | 72.19 | 30.86 | 0.17 |
| Hill | 36.42 | 73.82 | 31.76 | 0.16 |

**Table S11. The average mechanical properties of $Li_{12}Si_7$ calculated by Voigt-Reuss-Hill approximation.**

| | | | | | |
|---|---|---|---|---|---|
| 86.72 | 27.07 | -1.45 | 0 | 0 | 7.45 |
| 27.07 | 83.64 | 1.15 | 0 | 0 | -8.77 |
| -1.45 | 1.15 | 147.70 | 0 | 0 | 2.50 |
| 0 | 0 | 0 | 30.82 | -12.51 | 0 |
| 0 | 0 | 0 | -12.51 | 24.64 | 0 |
| 7.45 | -8.77 | 2.50 | 0 | 0 | 20.64 |

**Table S12. The calculated stiffness matrix (coefficients in GPa) of $Li_7Si_3$.**

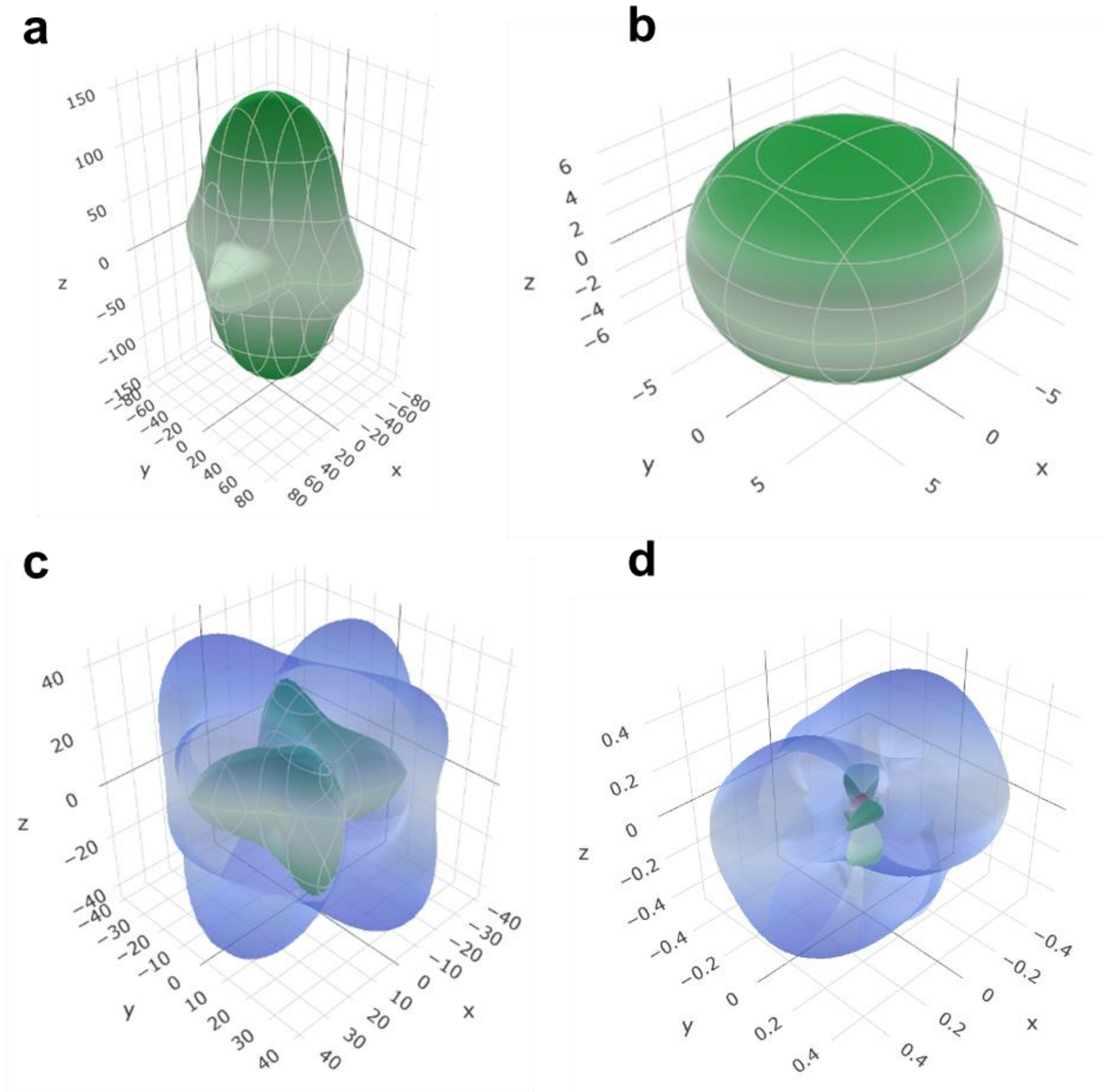


**Fig. S26. Calculated mechanical properties of $Li_7Si_3$.** (a) Spatial dependence of the Young's modulus. (b) Spatial dependence of linear compressibility. (c) Spatial dependence of the shear modulus. (d) Spatial dependence of the Poisson's ratio.

| **Averaging scheme** | **Bulk modulus (GPa)** | **Young's modulus (GPa)** | **Shear modulus (GPa)** | **Poisson's ratio** |
|---|---|---|---|---|
| Voigt | 41.29 | 81.21 | 34.64 | 0.17 |
| Reuss | 40.61 | 62.14 | 24.96 | 0.24 |
| Hill | 40.95 | 71.94 | 29.80 | 0.21 |

**Table S13. The average mechanical properties of $Li_7Si_3$ calculated by Voigt-Reuss-Hill approximation.**

| | | | | | |
|---|---|---|---|---|---|
| 86.23 | 7.23 | 11.19 | 0 | 0 | 0 |
| 7.23 | 81.45 | 16.31 | 0 | 0 | 0 |
| 11.19 | 16.31 | 70.99 | 0 | 0 | 0 |
| 0 | 0 | 0 | 24.69 | 0 | 0 |
| 0 | 0 | 0 | 0 | 28.82 | 0 |
| 0 | 0 | 0 | 0 | 0 | 23.76 |

**Table S14. The calculated stiffness matrix (coefficients in GPa) of $Li_{13}Si_4$.**

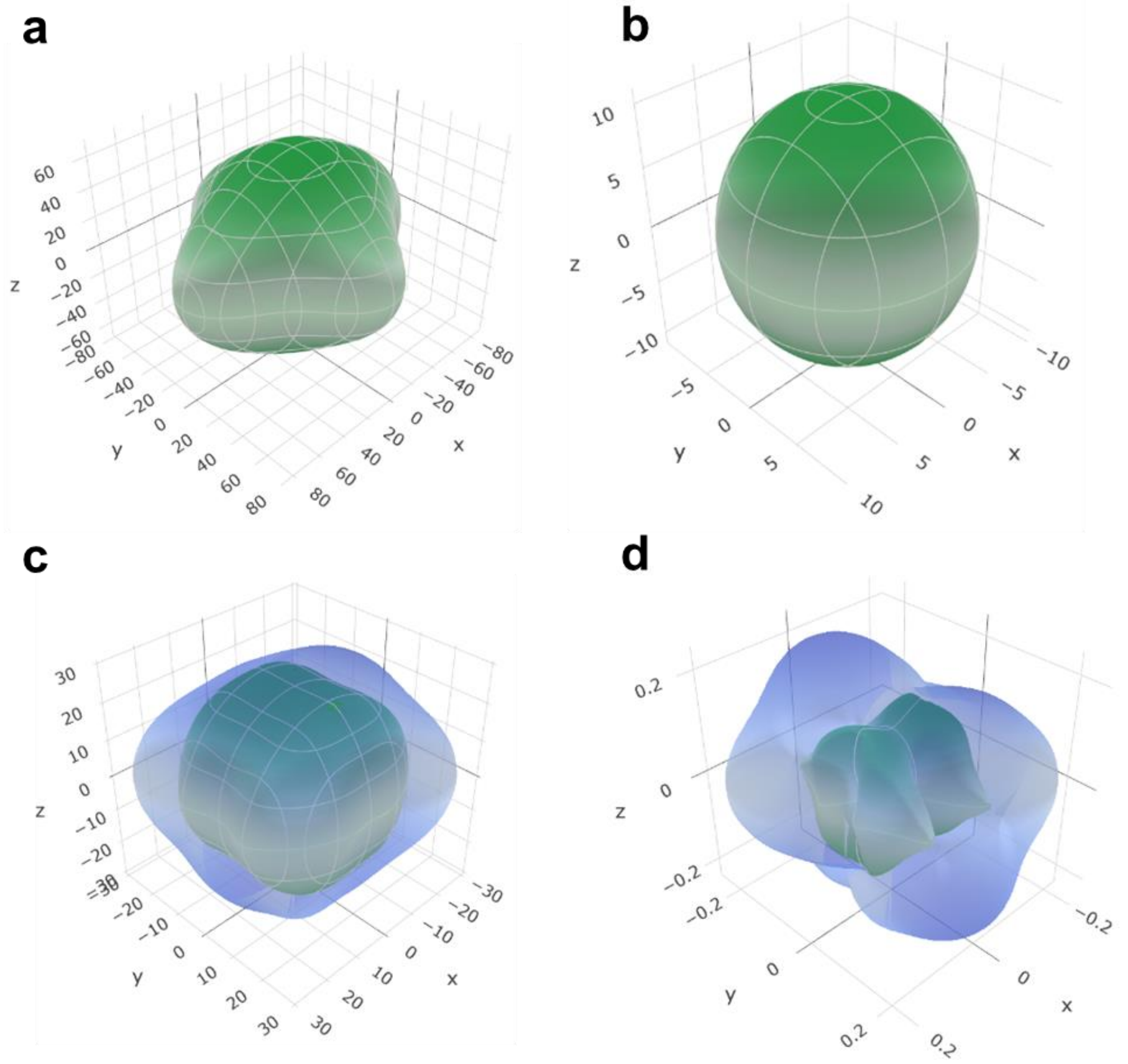


**Fig. S27. Calculated mechanical properties of $Li_{13}Si_4$.** (a) Spatial dependence of the Young's modulus. (b) Spatial dependence of linear compressibility. (c) Spatial dependence of the shear modulus. (d) Spatial dependence of the Poisson's ratio.

| Averaging scheme | Bulk modulus (GPa) | Young's modulus (GPa) | Shear modulus (GPa) | Poisson's ratio |
|---|---|---|---|---|
| Voigt | 34.24 | 67.94 | 29.05 | 0.17 |
| Reuss | 34.19 | 66.33 | 28.19 | 0.18 |
| Hill | 34.21 | 67.14 | 28.62 | 0.17 |

**Table S15. The average mechanical properties of $Li_{13}Si_4$ calculated by the Voigt-Reuss-Hill approximation.**

| | | | | | |
|---|---|---|---|---|---|
| 48.42 | 23.45 | 23.45 | 0 | 0 | 0 |
| 23.45 | 48.42 | 23.45 | 0 | 0 | 0 |
| 23.45 | 23.45 | 48.42 | 0 | 0 | 0 |
| 0 | 0 | 0 | 32.91 | 0 | 0 |
| 0 | 0 | 0 | 0 | 32.91 | 0 |
| 0 | 0 | 0 | 0 | 0 | 32.91 |

**Table S16. The calculated stiffness matrix (coefficients in GPa) of $Li_{15}Si_4$.**

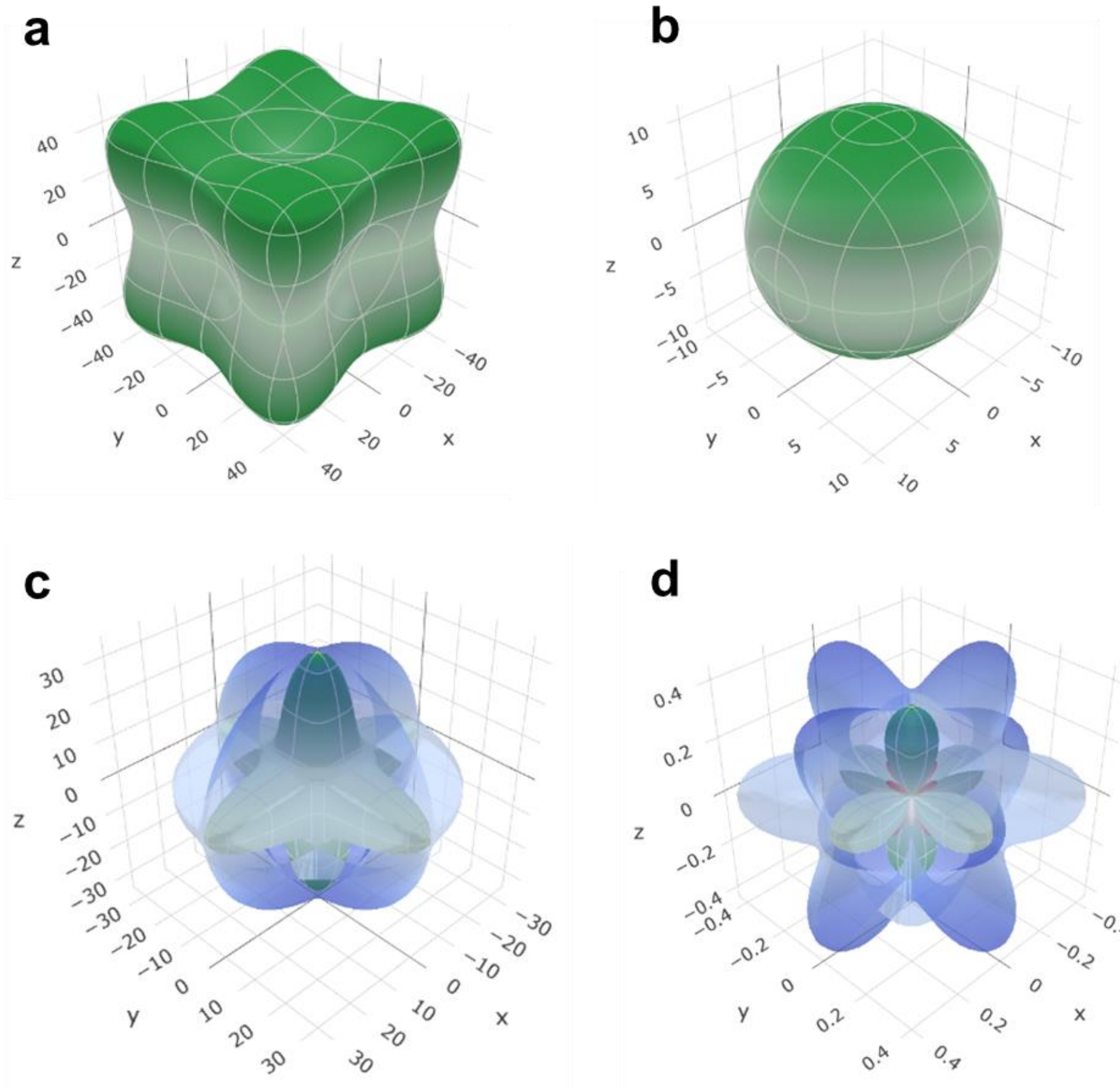


**Fig. S28. Calculated mechanical properties of $Li_{15}Si_4$.** (a) Spatial dependence of the Young's modulus. (b) Spatial dependence of linear compressibility. (c) Spatial dependence of the shear modulus. (d) Spatial dependence of the Poisson's ratio.

| Averaging scheme | Bulk modulus (GPa) | Young's modulus (GPa) | Shear modulus (GPa) | Poisson's ratio |
|---|---|---|---|---|
| Voigt | 31.77 | 58.92 | 24.74 | 0.19 |
| Reuss | 31.77 | 49.37 | 19.89 | 0.24 |
| Hill | 31.77 | 54.25 | 22.32 | 0.22 |

**Table S17. The average mechanical properties of $Li_{15}Si_4$ calculated by the Voigt-Reuss-Hill approximation.**

## Supplementary Discussion 2: DMT modulus of Li-Si alloy anodes

To systematically investigate the modulus evolution of Li-Si alloy anodes, we synthesized a series of alloys with varying Li/Si ratios and characterized their mechanical properties using AFM-based Derjaguin-Muller-Toporov (DMT) modulus mapping. Our analysis reveals a clear trend of mechanical softening with increasing Li content: $Li_{0.2}Si$ 3.0 GPa (Fig. S29), $Li_{0.5}Si$ 2.9 GPa (Fig. S30), LiSi 2.7 GPa (Fig. S31), $Li_{12}Si_7$ ($Li_{1.71}Si$) 2.1 GPa (Fig. S32), $Li_7Si_3$ ($Li_{2.33}Si$) 1.8 GPa (Fig. S33), $Li_{13}Si_4$ ($Li_{3.25}Si$) 1.5 GPa (Fig. S34) and $Li_{15}Si_4$ ($Li_{3.75}Si$) 1.2 GPa (Fig. 3D-E).The comprehensive modulus data across all compositions (summarized in Fig. S35 and Table S18) demonstrate a consistent 60% reduction in mechanical stiffness from the least lithiated ($Li_{0.2}Si$) to the most lithiated ($Li_{15}Si_4$) phase. This progressive softening behavior, visualized through correlated AFM topography and modulus mapping (Figs. 3D-E, S29-S35), suggests that the modulus ratio between anode and solid-state electrolyte (SSE) can be effectively tuned by controlling the lithiation degree of Si-based anodes, offering a potential strategy for mitigating interfacial stresses in all-solid-state batteries.

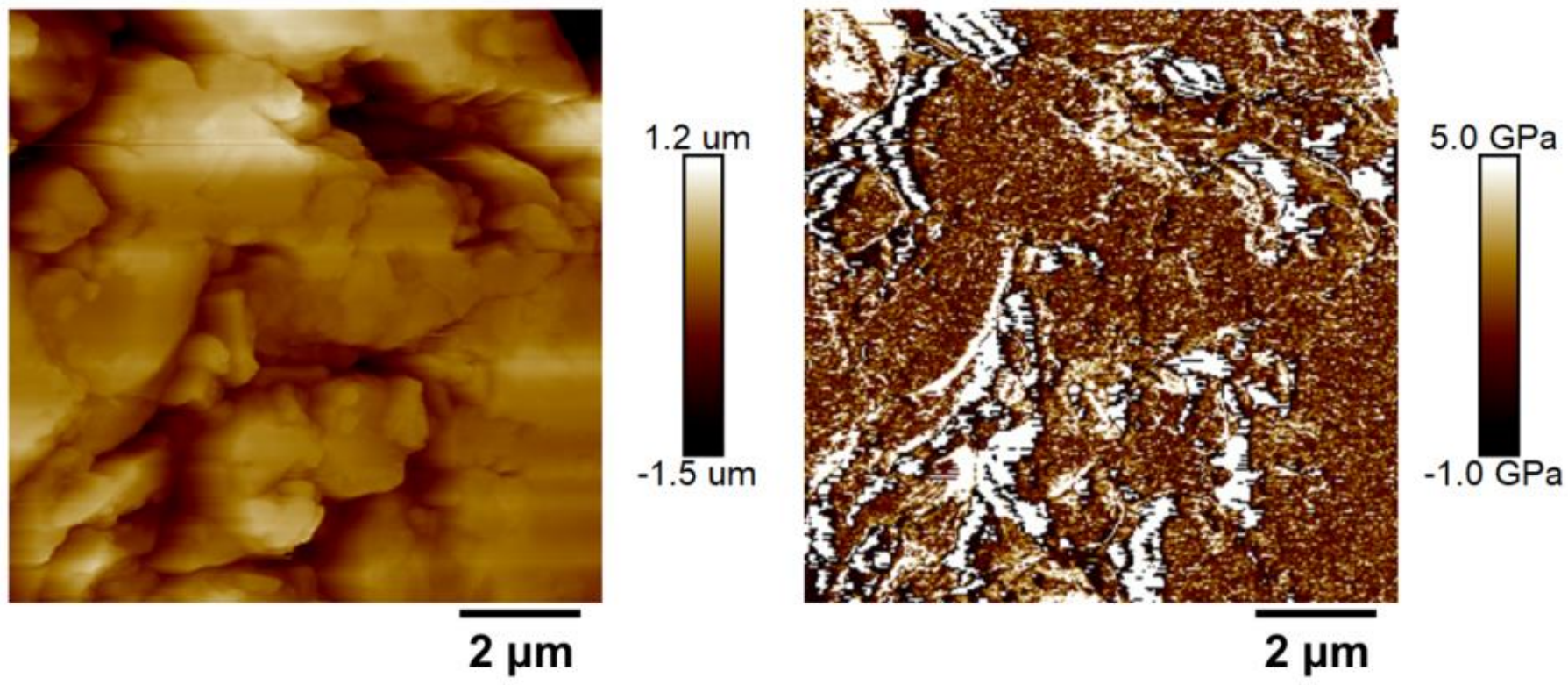


**Fig. S29. AFM image and related modulus image of $Li_{0.2}Si$ alloy material.**

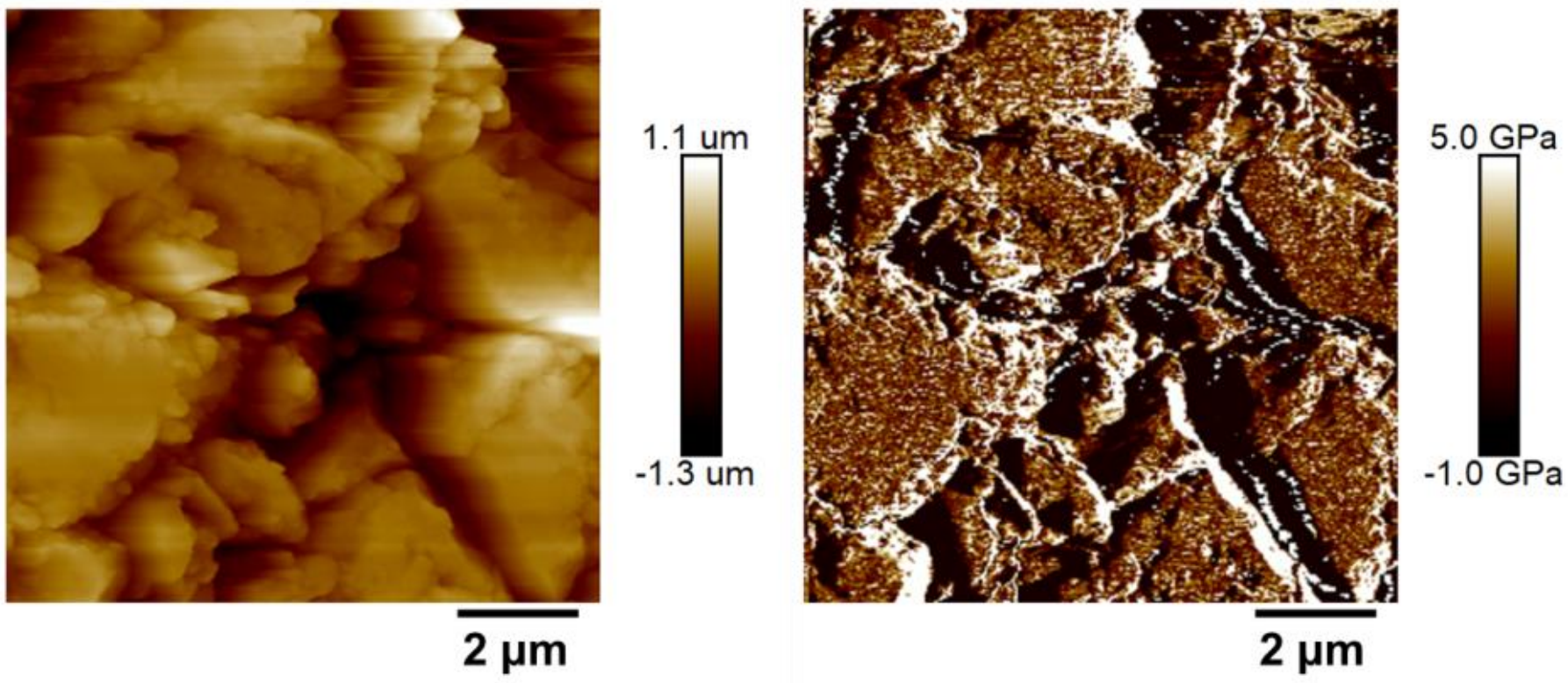


**Fig. S30. AFM image and related modulus image of $Li_{0.5}Si$ alloy material.**

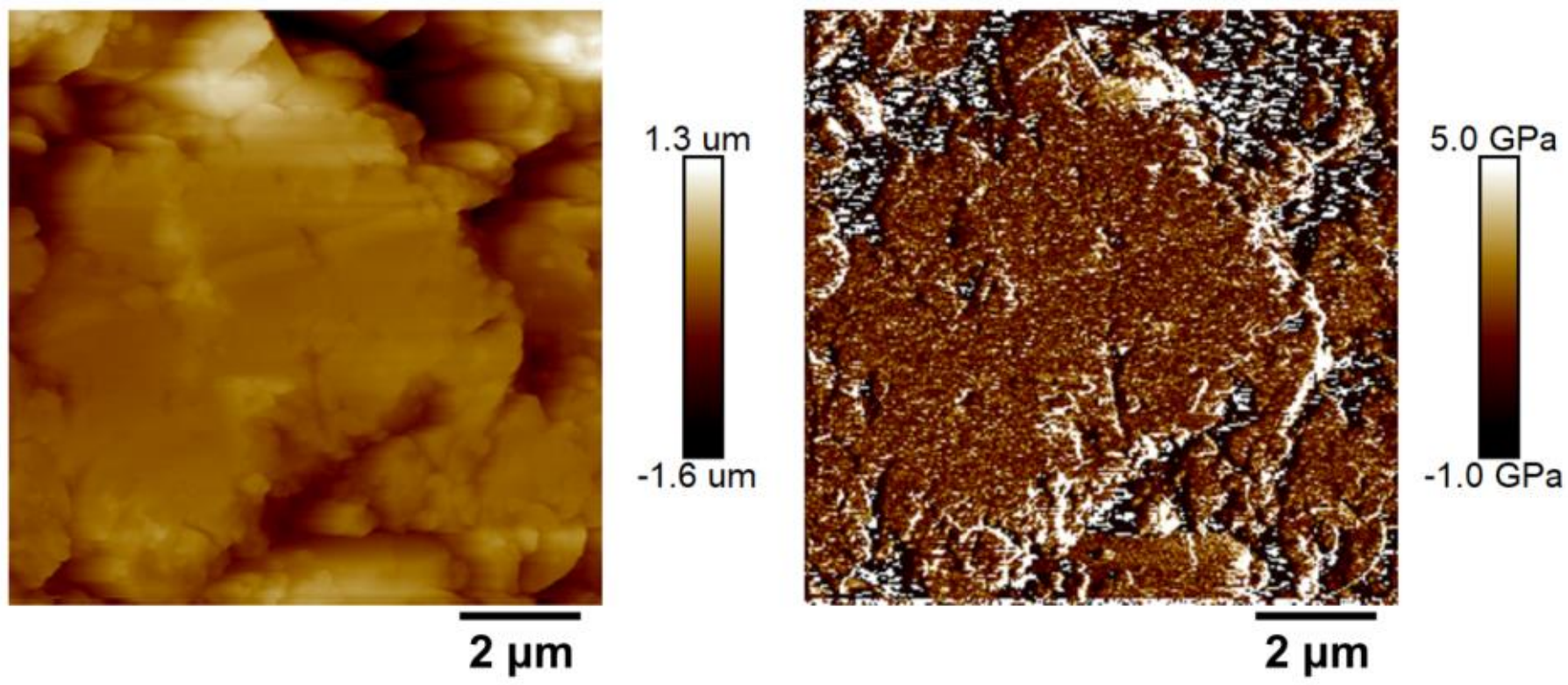


**Fig. S31. AFM image and related modulus image of LiSi alloy material.**

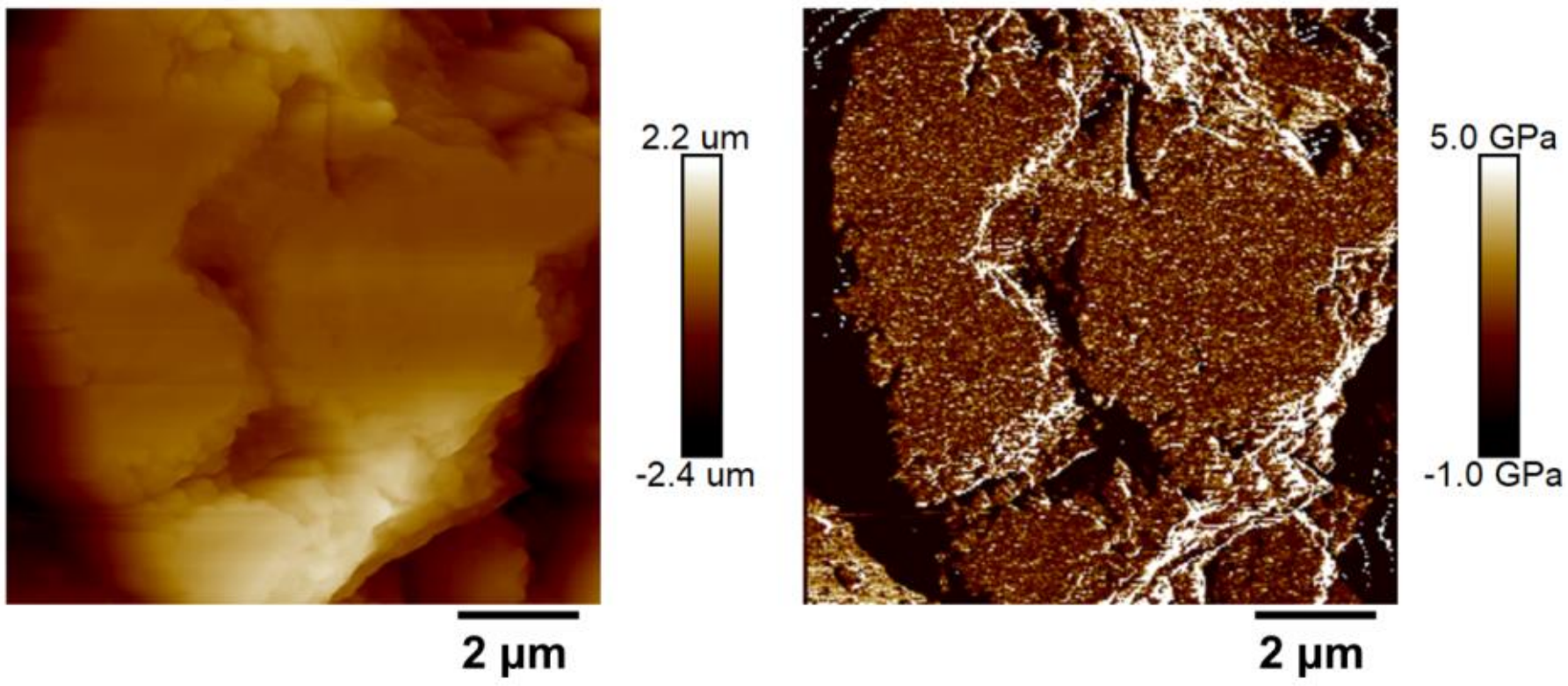


**Fig. S32. AFM image and related modulus image of $Li_{12}Si_7$ ($Li_{1.71}Si$) alloy material.**

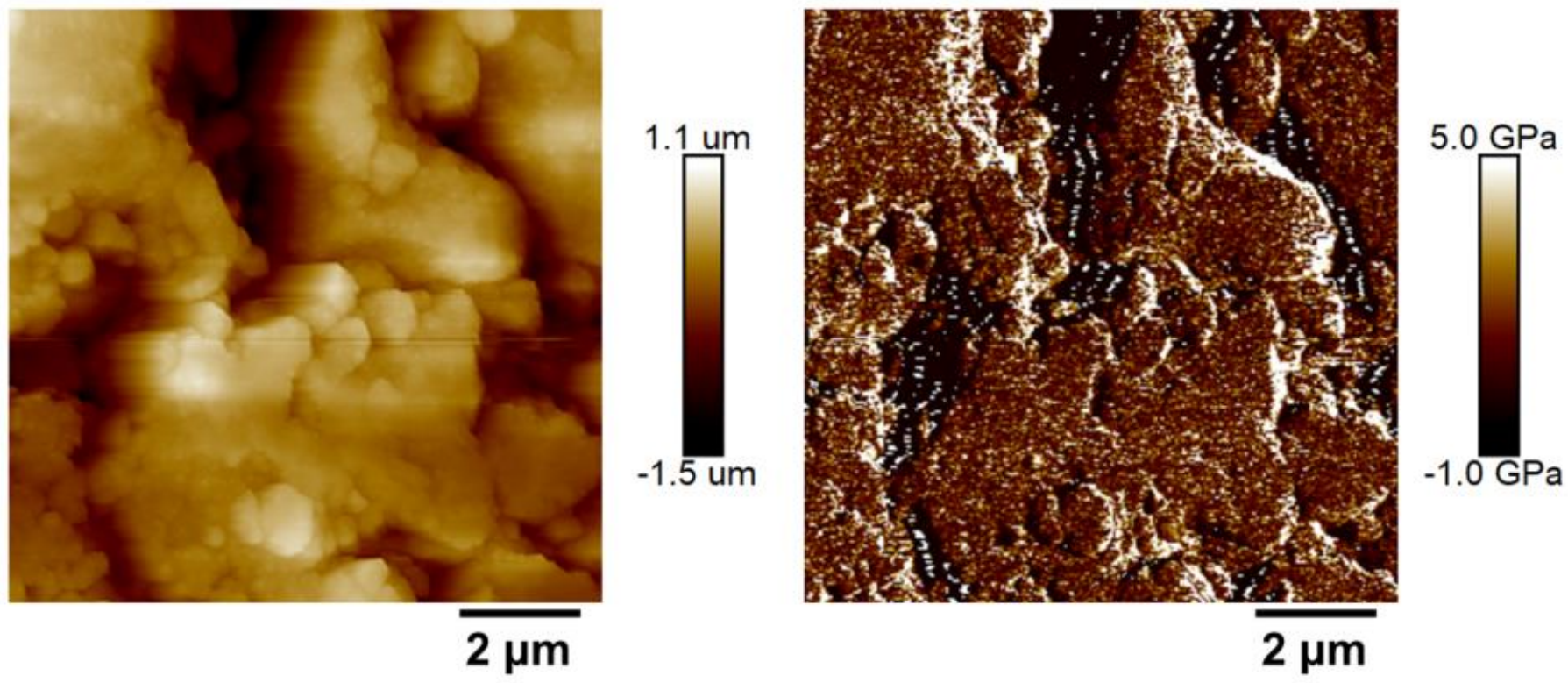


**Fig. S33. AFM image and related modulus image of $Li_7Si_3$ ($Li_{2.33}Si$) alloy material.**

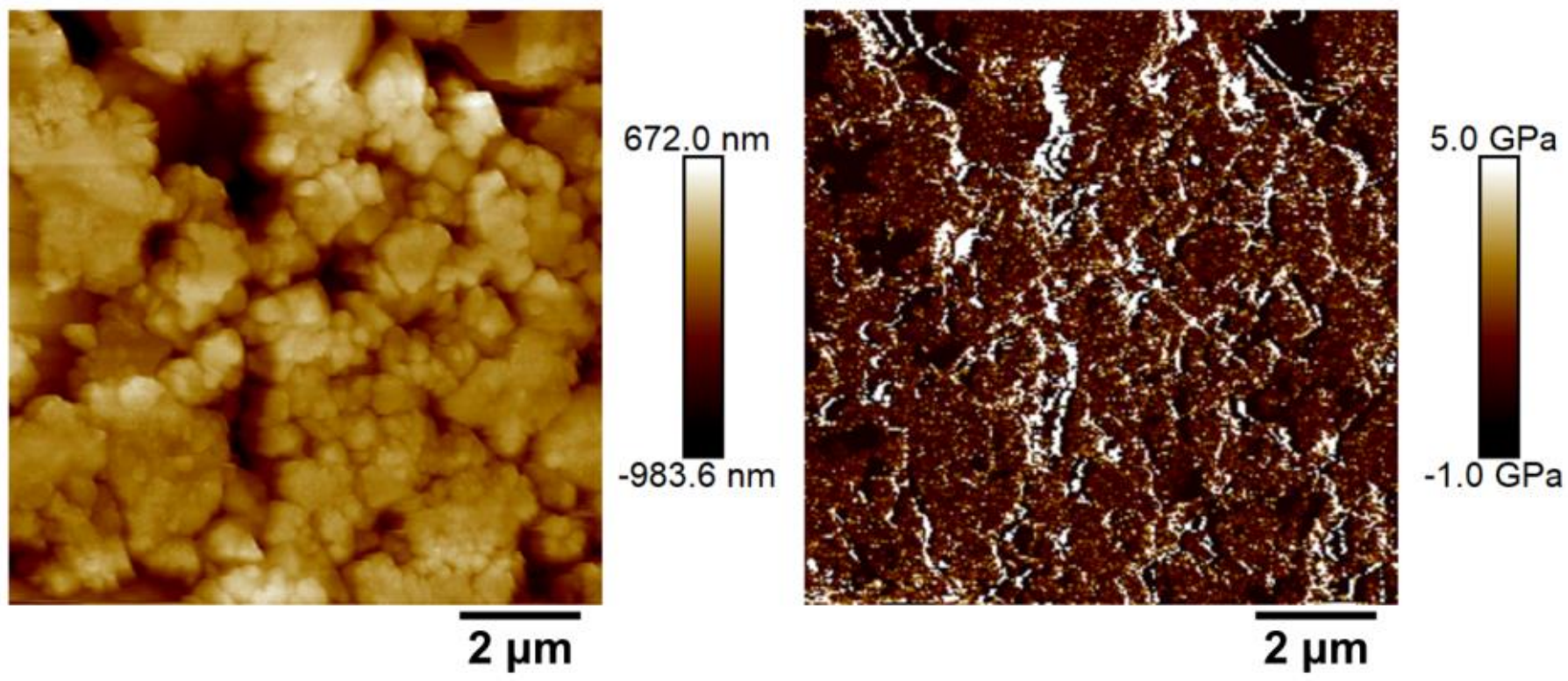


**Fig. S34. AFM image and related modulus image of $Li_{13}Si_4$ ($Li_{3.25}Si$) alloy material.**

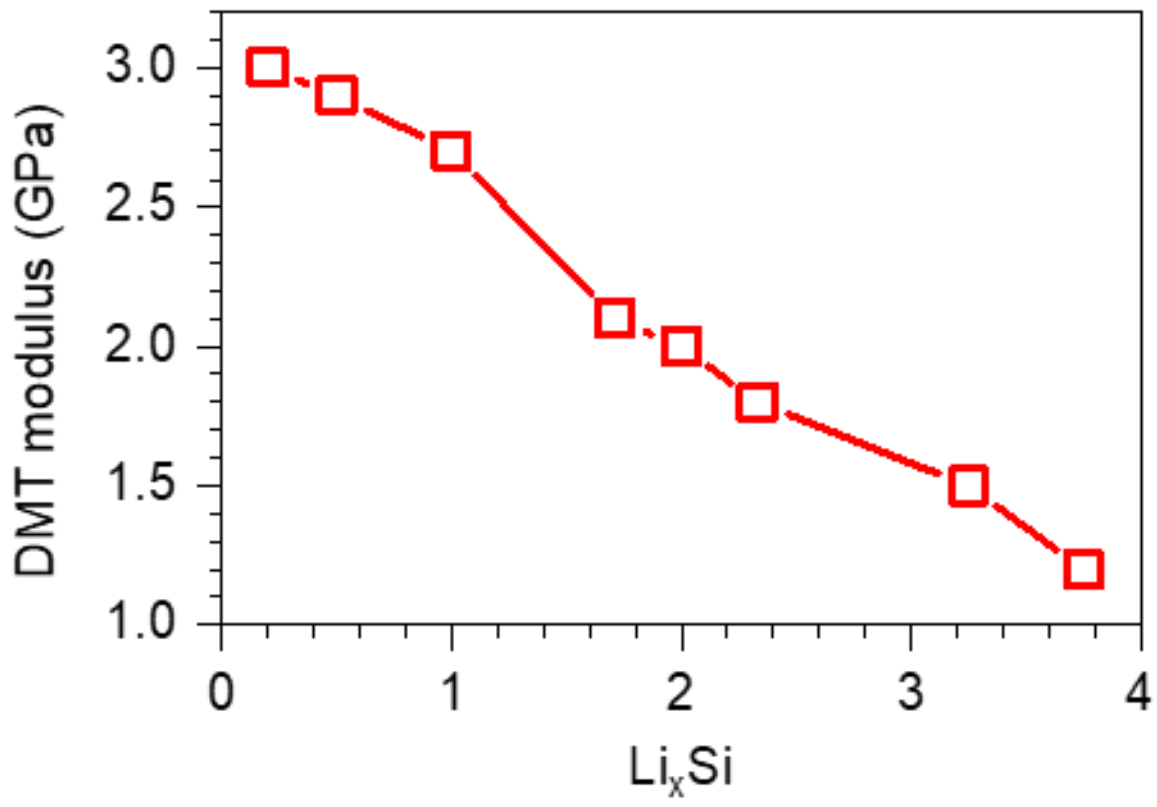


**Fig. S35. The Young's Modulus of Li-Si alloy with different Li contents using the Derjaguin-Muller-Toporov (DMT) model.**

| Components | DMT modulus (GPa) |
|---|---|
| $Li_{0.2}Si$ | 3.0 |
| $Li_{0.5}Si$ | 2.9 |
| LiSi | 2.7 |
| $Li_{12}Si_7$ ($Li_{1.71}Si$) | 2.1 |
| $Li_7Si_3$ ($Li_{2.33}Si$) | 1.8 |
| $Li_{13}Si_4$ ($Li_{3.25}Si$) | 1.5 |
| $Li_{15}Si_4$ ($Li_{3.75}Si$) | 1.2 |

**Table S18. The DMT modulus of Li-Si alloy with different Li contents.**

**REFERENCES**


1. A. Manthiram, X. Yu, S. Wang, Lithium battery chemistries enabled by solid-state electrolytes. *Nat. Rev. Mater.* **2**, 16103 (2017).

2. J. Fu, C. Wang, S. Wang, J. W. Reid, J. Liang, J. Luo, J. T. Kim, Y. Zhao, X. Yang, F. Zhao, W. Li, B. Fu, X. Lin, Y. Hu, H. Su, X. Hao, Y. Gao, S. Zhang, Z. Wang, J. Liu, H. Abdolvand, T. K. Sham, Y. Mo, X. Sun, A cost-effective all-in-one halide material for all-solid-state batteries. *Nature* **643**, 111–118 (2025).

3. Q. Zhao, S. Stalin, C.-Z. Zhao, L. A. Archer, Designing solid-state electrolytes for safe, energy-dense batteries. *Nat. Rev. Mater.* **5**, 229–252 (2020).

4. H. Song, K. Munch, X. Liu, K. Shen, R. Zhang, T. Weintraut, Y. Yusim, D. Jiang, X. Hong, J. Meng, Y. Liu, M. He, Y. Li, P. Henkel, T. Brezesinski, J. Janek, Q. Pang, All-solid-state Li-S batteries with fast solid-solid sulfur reaction. *Nature* **637**, 846–853 (2025).

5. D. H. S. Tan, Y. T. Chen, H. D. Yang, W. Bao, B. Sreenarayanan, J. M. Doux, W. K. Li, B. Y. Lu, S. Y. Ham, B. Sayahpour, J. Scharf, E. A. Wu, G. Deysher, H. E. Han, H. J. Hah, H. Jeong, J. B. Lee, Z. Chen, Y. S. Meng, Carbon-free high-loading silicon anodes enabled by sulfide solid electrolytes. *Science* **373**, 1494–1499 (2021).

6. D. Cao, X. Sun, Y. Li, A. Anderson, W. Lu, H. Zhu, Long-cycling sulfide-based all-solid-state batteries enabled by electrochemo-mechanically stable electrodes. *Adv. Mater.* **34**, e2200401 (2022).

7. X. Zhan, M. Li, S. Li, X. Pang, F. Mao, H. Wang, Z. Sun, X. Han, B. Jiang, Y.-B. He, M. Li, Q. Zhang, L. Zhang, Challenges and opportunities towards silicon-based all-solid-state batteries. *Energy Storage Mater.* **61**, 102875 (2023).

8. X. H. Liu, J. Y. Huang, *In situ* TEM electrochemistry of anode materials in lithium ion batteries. *Energ. Environ. Sci.* **4**, 3844–3860 (2011).

9. L. Yang, H.-S. Chen, H. Jiang, Y.-J. Wei, W.-L. Song, D.-N. Fang, Failure mechanisms of 2D silicon film anodes: In situ observations and simulations on crack evolution. *Chem. Commun.* **54**, 3997–4000 (2018).

10. H. Wu, G. Chan, J. W. Choi, I. Ryu, Y. Yao, M. T. McDowell, S. W. Lee, A. Jackson, Y. Yang, L. Hu, Y. Cui, Stable cycling of double-walled silicon nanotube battery anodes through solid-electrolyte interphase control. *Nat. Nanotechnol.* **7**, 310–315 (2012).

11. Y. He, L. Jiang, T. Chen, Y. Xu, H. Jia, R. Yi, D. Xue, M. Song, A. Genc, C. Bouchet-Marquis, L. Pullan, T. Tessner, J. Yoo, X. Li, J.-G. Zhang, S. Zhang, C. Wang, Progressive growth of the solid-electrolyte interphase towards the Si anode interior causes capacity fading. *Nat. Nanotechnol.* **16**, 1113–1120 (2021).

12. S. Kalnaus, N. J. Dudney, A. S. Westover, E. Herbert, S. Hackney, Solid-state batteries: The critical role of mechanics. *Science* **381**, eabg5998 (2023).

13. J. Fan, C. Liu, N. Li, L. Yang, X. G. Yang, B. Dou, S. Hou, X. Feng, H. Jiang, H. Li, W. L. Song, L. Sun, H. S. Chen, H. Gao, D. Fang, Wireless transmission of internal hazard signals in Li-ion batteries. *Nature* **641**, 639–645 (2025).

14. V. A. Sethuraman, M. J. Chon, M. Shimshak, V. Srinivasan, P. R. Guduru, *In situ* measurements of stress evolution in silicon thin films during electrochemical lithiation and delithiation. *J. Power Sources* **195**, 5062–5066 (2010).

15. L. Porz, T. Swamy, B. W. Sheldon, D. Rettenwander, T. Frömling, H. L. Thaman, S. Berendts, R. Uecker, W. C. Carter, Y.-M. Chiang, Mechanism of lithium metal penetration through inorganic solid electrolytes. *Adv. Energy Mater.* **7**, 1701003 (2017).

16. E. Kazyak, R. Garcia-Mendez, W. S. LePage, A. Sharafi, A. L. Davis, A. J. Sanchez, K.-H. Chen, C. Haslam, J. Sakamoto, N. P. Dasgupta, Li penetration in ceramic solid electrolytes: Operando microscopy analysis of morphology, propagation, and reversibility. *Matter* **2**, 1025–1048 (2020).

17. Z. Ning, D. S. Jolly, G. Li, R. De Meyere, S. D. Pu, Y. Chen, J. Kasemchainan, J. Ihli, C. Gong, B. Liu, D. L. R. Melvin, A. Bonnin, O. Magdysyuk, P. Adamson, G. O. Hartley, C. W. Monroe, T. J. Marrow, P. G. Bruce, Visualizing plating-induced cracking in lithium-anode solid-electrolyte cells. *Nat. Mater.* **20**, 1121–1129 (2021).

18. M. T. McDowell, S. W. Lee, W. D. Nix, Y. Cui, Understanding the lithiation of silicon and other alloying anodes for lithium-ion batteries. *Adv. Mater.* **25**, 4966–4985 (2013).

19. H. Kim, C.-Y. Chou, J. G. Ekerdt, G. S. Hwang, Structure and properties of Li-Si alloys: A first-principles study. *J. Phys. Chem. C* **115,** 2514–2521 (2011).

20. H. Huo, M. Jiang, Y. Bai, S. Ahmed, K. Volz, H. Hartmann, A. Henss, C. V. Singh, D. Raabe, J. Janek, Chemo-mechanical failure mechanisms of the silicon anode in solid-state batteries. *Nat. Mater.* **23**, 543–551 (2024).

21. X. Gao, X. Yang, K. Adair, J. Liang, Q. Sun, Y. Zhao, R. Li, T. K. Sham, X. Sun, Fast charging all solid-state lithium batteries enabled by rational design of dual vertically-aligned electrodes. *Adv. Funct. Mater.* **30**, 2005357 (2020).

22. Y.-G. Lee, S. Fujiki, C. Jung, N. Suzuki, N. Yashiro, R. Omoda, D.-S. Ko, T. Shiratsuchi, T. Sugimoto, S. Ryu, J. H. Ku, T. Watanabe, Y. Park, Y. Aihara, D. Im, I. T. Han, High-energy long-cycling all-solid-state lithium metal batteries enabled by silver-carbon composite anodes. *Nat. Energy* **5**, 299–308 (2020).

23. J. B. Park, C. Choi, S. Yu, K. Y. Chung, D. W. Kim, Porous lithiophilic Li-Si alloy-type interfacial framework via self-discharge mechanism for stable lithium metal anode. *Adv. Energy Mater.* **11**, 2101544 (2021).

24. L. Ye, X. Li, A dynamic stability design strategy for lithium metal solid state batteries. *Nature* **593**, 218–222 (2021).

25. M. J. Lee, J. Han, K. Lee, Y. J. Lee, B. G. Kim, K. N. Jung, B. J. Kim, S. W. Lee, Elastomeric electrolytes for high-energy solid-state lithium batteries. *Nature* **601**, 217–222 (2022).

26. S. Sun, C. Z. Zhao, H. Yuan, Z. H. Fu, X. Chen, Y. Lu, Y. F. Li, J. K. Hu, J. Dong, J. Q. Huang, M. Ouyang, Q. Zhang, Eliminating interfacial O-involving degradation in

Li-rich Mn-based cathodes for all-solid-state lithium batteries. *Sci. Adv.* **8**, eadd5189 (2022).

27. L. Zhou, T.-T. Zuo, C. Y. Kwok, S. Y. Kim, A. Assoud, Q. Zhang, J. Janek, L. F. Nazar, High areal capacity, long cycle life 4 V ceramic all-solid-state Li-ion batteries enabled by chloride solid electrolytes. *Nat. Energy* **7**, 83–93 (2022).

28. Y. C. Yin, J. T. Yang, J. D. Luo, G. X. Lu, Z. Huang, J. P. Wang, P. Li, F. Li, Y. C. Wu, T. Tian, Y. F. Meng, H. S. Mo, Y. H. Song, J. N. Yang, L. Z. Feng, T. Ma, W. Wen, K. Gong, L. J. Wang, H. X. Ju, Y. Xiao, Z. Li, X. Tao, H. B. Yao, A $LaCl_3$-based lithium superionic conductor compatible with lithium metal. *Nature* **616**, 77–83 (2023).

29. S. Y. Ham, E. Sebti, A. Cronk, T. Pennebaker, G. Deysher, Y. T. Chen, J. A. S. Oh, J. B. Lee, M. S. Song, P. Ridley, D. H. S. Tan, R. J. Clement, J. Jang, Y. S. Meng, Overcoming low initial coulombic efficiencies of Si anodes through prelithiation in all-solid-state batteries. *Nat. Commun.* **15**, 2991 (2024).

30. S. Jing, Y. Lu, Y. Huang, H. Liu, Y. Shen, W. Kuang, H. Shen, S. Liu, Z. Zhang, F. Liu, High-performance sheet-type sulfide all-solid-state batteries enabled by dual-function $Li_{4.4}Si$ alloy-modified nano silicon anodes. *Adv. Mater.* **36**, 2312305 (2024).

31. Z. T. Liu, J. Liu, S. M. Zhao, S. N. Xun, P. Byaruhanga, S. Chen, Y. Z. Tang, T. Zhu, H. L. Chen, Low-cost iron trichloride cathode for all-solid-state lithium-ion batteries. *Nat. Sustain.* **7**, 1492–1500 (2024).

32. H. Pan, L. Wang, Y. Shi, C. Sheng, S. Yang, P. He, H. Zhou, A solid-state lithium-ion battery with micron-sized silicon anode operating free from external pressure. *Nat. Commun.* **15**, 2263 (2024).

33. H. Su, J. Li, Y. Zhong, Y. Liu, X. Gao, J. Kuang, M. Wang, C. Lin, X. Wang, J. Tu, A scalable Li-Al-Cl stratified structure for stable all-solid-state lithium metal batteries. *Nat. Commun.* **15**, 4202 (2024).

34. H. Su, Y. Zhong, C. Wang, Y. Liu, Y. Hu, J. Li, M. Wang, L. Jiao, N. Zhou, B. Xiao, X. Wang, X. Sun, J. Tu, Deciphering the critical role of interstitial volume in glassy sulfide superionic conductors. *Nat. Commun.* **15**, 2552 (2024).

35. L. Wang, A. Mukherjee, C. Y. Kuo, S. Chakrabarty, R. Yemini, A. A. Dameron, J. W. DuMont, S. H. Akella, A. Saha, S. Taragin, H. Aviv, D. Naveh, D. Sharon, T. S. Chan, H. J. Lin, J. F. Lee, C. T. Chen, B. Liu, X. Gao, S. Basu, Z. Hu, D. Aurbach, P. G. Bruce, High-energy all-solid-state lithium batteries enabled by Co-free $LiNiO_2$ cathodes with robust outside-in structures. *Nat. Nanotechnol.* **19**, 208–218 (2024).

36. Z. Zhang, Z. Sun, X. Han, Y. Liu, S. Pei, Y. Li, L. Luo, P. Su, C. Lan, Z. Zhang, S. Xu, S. Guo, W. Huang, S. Chen, M.-S. Wang, An all-electrochem-active silicon anode enabled by spontaneous Li-Si alloying for ultra-high performance solid-state batteries. *Energ. Environ. Sci.* **17**, 1234–1245 (2024).

37. J. Zhou, M. L. Holekevi Chandrappa, S. Tan, S. Wang, C. Wu, H. Nguyen, C. Wang, H. Liu, S. Yu, Q. R. S. Miller, G. Hyun, J. Holoubek, J. Hong, Y. Xiao, C. Soulen, Z. Fan, E. E. Fullerton, C. J. Brooks, C. Wang, R. J. Clement, Y. Yao, E. Hu, S. P. Ong,

P. Liu, Healable and conductive sulfur iodide for solid-state Li-S batteries. *Nature* **627**, 301–305 (2024).

38. R. Gaillac, P. Pullumbi, F. X. Coudert, ELATE: An open-source online application for analysis and visualization of elastic tensors. *J. Phys. Condens. Matter* **28**, 275201 (2016).

39. J. Wen, Y. Wei, Y.-T. Cheng, Stress evolution in elastic-plastic electrodes during electrochemical processes: A numerical method and its applications. *J. Mech. Phys. Solids* **116**, 403–415 (2018).

40. J. Wen, Y. Wei, Y.-T. Cheng, Examining the validity of Stoney-equation for in-situ stress measurements in thin film electrodes using a large-deformation finite-element procedure. *J. Power Sources* **387**, 126–134 (2018).

41. W. Yan, Z. Mu, Z. Wang, Y. Huang, D. Wu, P. Lu, J. Lu, J. Xu, Y. Wu, T. Ma, M. Yang, X. Zhu, Y. Xia, S. Shi, L. Chen, H. Li, F. Wu, Hard-carbon-stabilized Li-Si anodes for high-performance all-solid-state Li-ion batteries. *Nat. Energy* **8**, 800–813 (2023).

42. C. Li, Y. Wu, F. Ren, J. Liu, Y. Lin, M. Xia, H. Wang, C. Meng, Z. Wang, Z. Gong, Y. Yang, Pre-lithiated silicon-based composite anode for high-performance all-solid-state batteries. *Small* **21**, e2411451 (2025).
